# Reproductive Aging in *Caenorhabditis elegans*: From Molecules to Ecology

**DOI:** 10.3389/fcell.2021.718522

**Published:** 2021-09-16

**Authors:** Andrea Scharf, Franziska Pohl, Brian M. Egan, Zuzana Kocsisova, Kerry Kornfeld

**Affiliations:** ^1^Department of Developmental Biology, Washington University School of Medicine, St. Louis, MO, United States; ^2^Department of Medicine, Washington University School of Medicine, St. Louis, MO, United States

**Keywords:** reproduction, germ line, aging, evolution, matricidal hatching, egg-laying, oocyte quality, menopause

## Abstract

Aging animals display a broad range of progressive degenerative changes, and one of the most fascinating is the decline of female reproductive function. In the model organism *Caenorhabditis elegans*, hermaphrodites reach a peak of progeny production on day 2 of adulthood and then display a rapid decline; progeny production typically ends by day 8 of adulthood. Since animals typically survive until day 15 of adulthood, there is a substantial post reproductive lifespan. Here we review the molecular and cellular changes that occur during reproductive aging, including reductions in stem cell number and activity, slowing meiotic progression, diminished Notch signaling, and deterioration of germ line and oocyte morphology. Several interventions have been identified that delay reproductive aging, including mutations, drugs and environmental factors such as temperature. The detailed description of reproductive aging coupled with interventions that delay this process have made *C. elegans* a leading model system to understand the mechanisms that drive reproductive aging. While reproductive aging has dramatic consequences for individual fertility, it also has consequences for the ecology of the population. Population dynamics are driven by birth and death, and reproductive aging is one important factor that influences birth rate. A variety of theories have been advanced to explain why reproductive aging occurs and how it has been sculpted during evolution. Here we summarize these theories and discuss the utility of *C. elegans* for testing mechanistic and evolutionary models of reproductive aging.

## Introduction

Aging can be defined as the progressive decline of tissue morphology and function with increasing chronological age that eventually results in death of the organism. Since the discovery of single gene mutations that delay age-related decline and extend lifespan, *Caenorhabditis elegans* has become one of the leading model organisms to study aging (Friedman and Johnson, 1988; Kenyon et al., 1993; Tissenbaum, 2015). This non-parasitic, 1 mm nematode species lives on rotten plant material in the wild and can be easily cultivated on agar dishes or liquid medium in the laboratory (Corsi et al., 2015). *C. elegans* is well suited for aging research due to its short average lifespan of approximately 15 days and well characterized progressive, degenerative changes that are also observed in many larger animals with much longer lifespans, such as mammals (Collins et al., 2007). Interestingly, the nematode also experiences a loss of reproductive capacity in mid-life and a substantial post reproductive lifespan, similar to human females that undergo menopause around 50 years of age and typically survive until 80 years of age. In addition, oocyte quality decreases during hermaphrodite aging, which parallels the increasing likelihood of birth defects with increasing maternal age in humans (Nagaoka et al., 2012). These similarities between nematodes and humans indicate that *C. elegans* is a relevant model system to investigate the process of reproductive aging. The major advantage of *C. elegans* is that it is an experimentally powerful model organism: the animals are easy to cultivate, they are transparent which makes it easy to analyze morphological changes, and they are amenable to sophisticated genetic approaches due to their androdioecy (Morsci et al., 2011; Corsi et al., 2015). Populations consist of mostly hermaphrodites with few males, and they can be kept as clonal populations. The experimental advantages of studying this animal have led to systematic descriptions of age-related changes in its germ line, investigations of the underlying mechanisms, and the discovery of interventions that prolong the reproductive period and conserve oocyte quality later in life.

In this review, we focus on reproductive aging of the *C. elegans* hermaphrodite. Although males are of interest, it is much more difficult to analyze the age-related decline of male reproduction and little information is available. The germline of *C. elegans* hermaphrodites is regulated by sperm, and in hermaphrodites that lack sperm the oocytes display time-dependent loss of viability (Andux and Ellis, 2008; Adam Bohnert and Kenyon, 2017; Achache et al., 2021). We do not focus on this process because the regulatory circuit appears to function in young and old animals and thus is distinct from age-related degeneration. More specifically, we discuss observational studies that document the age-related decline of reproductive function in unmated and mated wild-type hermaphrodites, matricidal death as a reproductive aging phenotype, and morphological and molecular age-related changes of the reproductive organs. We then describe interventions that delay reproductive aging and provide evidence regarding mechanistic drivers, including single gene mutations, drugs, and environmental factors. Finally, we consider the ecological consequences of reproductive aging in light of evolutionary theory. Many other fine reviews cover the development and function of the germ line in young animals (Lints and Hall, 2009b; Hubbard and Schedl, 2019), the age-related decline of oocyte quality (Luo and Murphy, 2011), germ line soma interaction in aging (Antebi, 2013), and the comparison of reproductive aging in different model organisms including humans (Quesada-Candela et al., 2021).

**FIGURE 1:**
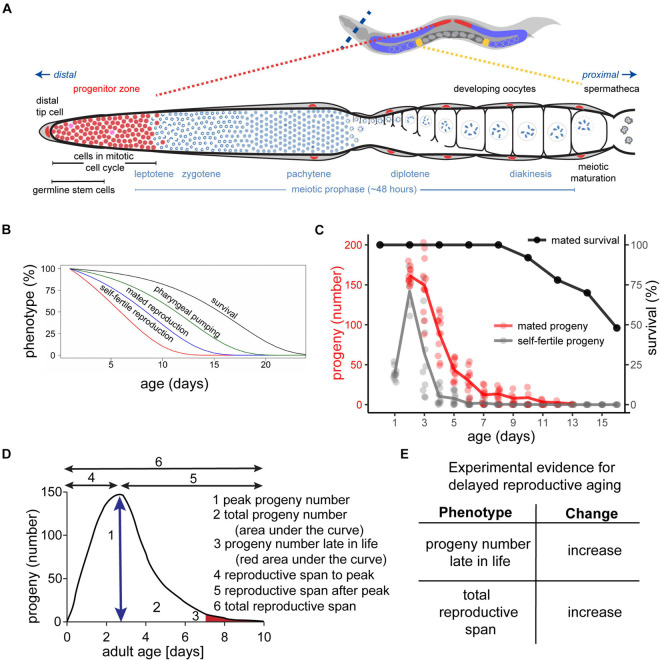
Somatic and reproductive aging in *Caenorhabditis elegans*. **(A)** Architecture of one of the two gonad arms of a young adult hermaphrodite. At the distal end of the gonad, the somatic distal tip cell (red) is embedded in other somatic gonad cells (gray) and maintains the germline stem cell fate via GLP-1/Notch signaling. The germline stem cells (SYGL-1 positive cells) progress from the distal end of the gonad to the proximal end. During this journey, they undergo mitosis in the progenitor zone (red), progress through the meiotic prophase and meiotic maturation. Finally, they are ready to be fertilized by sperm in the spermathecal (yellow). In adults, the progenitor zone and the early prophase of meiosis I overlap. Adapted from Kocsisova et al. (2019). **(B)** *C. elegans* exhibits age-related degenerative changes of the soma and the germ line. Self-fertile reproduction (red) and mated reproduction (blue) decreases before somatic functions such as pharyngeal pumping (green), and survival (black). Adapted from Kumar et al. (2014). **(C)** Progeny production curves of unmated/self-fertile (gray) versus mated (red) hermaphrodites. Mated hermaphrodites survive beyond their reproductive span (black). Note that hermaphrodites that died by matricidal hatching were censored. Adapted from Kocsisova et al. (2019). **(D)** Summary statistics for analysis of the progeny production curve: peak egg number (arrow under the curve); total egg number (area under total curve), egg number late in life (red area under tail of curve), reproductive span to the peak, reproductive span after the peak, and total reproductive span (black arrows). **(E)** Experimental evidence for delayed reproductive aging in mated hermaphrodites – an increase in the total reproductive span and/or an increase in the number of eggs laid late in life.

*Caenorhabditis elegans* develops from a fertilized egg into a mature egg-laying hermaphrodite in about 65 h at 20°C (Byerly et al., 1976). The reproductive organs are organized into two U-shaped symmetric gonad arms, through which developing eggs proceed in an assembly line fashion (Figure 1A). Each arm is comprised of the somatic gonad and the germ line, including the spermatheca. They unite in a shared uterus with the egg-laying apparatus. The function of the reproductive organs is to produce mature gametes, fertilize oocytes, and deposit the eggs into the environment. The stem cells reside in the progenitor zone of the distal syncytial germ line. The progenitor zone is about 20-cell-diameters long and harbors the only stem cells of adult *C. elegans*, the mitotic cycling germline stem cells. This region also includes the progenitor cells and the meiotic S-phase cells. Proliferation of germline stem cells is initiated and maintained by Notch ligand from the somatic distal tip cell. Germline stem cells that receive Notch signal remain in mitosis, but as they migrate proximally and lose contact with the distal tip cell, the stem cell nuclei enter the meiotic prophase with an average duration of 48 h including cellularization and growth (Hubbard and Schedl, 2019). It is challenging to precisely define these stem cells experimentally and conceptually, since they transition from the stem cell fate to a non-stem cell fate as they progress along the germ line. If stem cells are defined using molecular markers as SYGL-1 positive, then these cells do not proceed directly into meiotic S phase but rather they usually divide after losing SYGL-1 expression. In contrast to the early blastoderm in *Drosophila*, *C. elegans* stem cell nuclei are surrounded by cell membranes and behave like cells (Lints and Hall, 2009b). The number of germ cells that enter prophase of meiosis I is larger than the number that complete prophase of meiosis I as a result of apoptosis that occurs in late pachytene. The surviving germ cells then require an activation signal from the sperm in the form of the major sperm protein, which induces the completion of meiosis and the ovulation into the spermatheca, where fertilization occurs (Luo and Murphy, 2011; Pazdernik and Schedl, 2013; Huelgas-Morales and Greenstein, 2018; Hubbard and Schedl, 2019). After fertilization, the oocyte moves through the uterus and is pushed into the environment via the vulva. Production of fertilized eggs follows a time schedule that results in a characteristic progeny production curve (Figures 1C,D). After the hermaphrodite sexually matures, progeny production increases until it reaches a peak on adult day 2 (adult day 1 is defined as the 24-h period after the hermaphrodite laid its first egg, adult day 2 is defined as the subsequent 24-h period, etc.). At peak production, oocytes are ovulated every ∼23 min (McCarter et al., 1999), resulting in an average of ∼150 progeny per day. Progeny production rapidly decreases over the following days until it ceases around adult day 6–9. Summary statistics are useful to compare progeny production curves including timing of egg production (reproductive span to peak, reproductive span after peak, total reproductive span) and number of eggs produced (peak progeny number, total progeny number, progeny produced on day 7 and beyond) (Figures 1D,E).

## Reproductive Decline in Self-Fertile Versus Mated Hermaphrodites

Early studies by Klass (1977) and Croll et al. (1977) documented that there is an age-related peak and decline in fertilized eggs deposited into the environment that corresponds to the depletion of sperm in self-fertile hermaphrodites (Figures 1B,C). This is consistent with the findings of Ward and Carrel (1979) that there is an age-related decrease in the number of sperm in or near the spermatheca. As a result of sperm depletion, hermaphrodites begin to deposit unfertilized oocytes, resulting in a delayed, slightly overlapping, and much smaller peak compared to fertilized oocytes. Because the germ line responds to sperm depletion by arresting oocyte development, a phenotype called oocyte “stacking,” only a relatively small number of unfertilized oocytes are deposited. The term oocyte “stacking” refers to the columnar shape displayed by oocytes under these conditions. These age-related changes can be modulated by temperature, and the self-fertile reproductive span is extended by cold temperatures (Klass, 1977; Huang et al., 2004).

It is important to differentiate between self-fertile and mated reproduction. In self-fertile hermaphrodites, the first ∼300 germ cells differentiate as sperm, and the remaining germ cells differentiate as oocytes. As a consequence, the production of fertilized oocytes is limited by the number of self-sperm, and no additional self-sperm can be produced. Thus, the age-related decline of egg production displayed by self-fertile hermaphrodites is primarily a result of sperm depletion, and it is not necessarily an accurate indication of reproductive aging. Hermaphrodites can also mate with males, which are rare in the wild, but can be easily enriched under laboratory conditions (Lints and Hall, 2009a). Mating provides the hermaphrodites with excess sperm and removes the limitations of sperm depletion (Ward and Carrel, 1979; Hodgkin and Barnes, 1991). The difference between self-fertile and mated reproduction manifests in different reproductive spans and progeny numbers (Figures 1B,C). Wild-type hermaphrodites that are self-fertile display a total reproductive span of 4.6 days at 20°C on standard nematode growth medium seeded with live *E. coli* OP50. By contrast, a brief period of mating (24–48 h exposure to males) extends the total reproductive span to 8.8 days (Pickett et al., 2013). The total progeny number increases from ∼330 progeny in self-fertile hermaphrodites to ∼710 progeny in mated hermaphrodites (Pickett et al., 2013).

**FIGURE 2:**
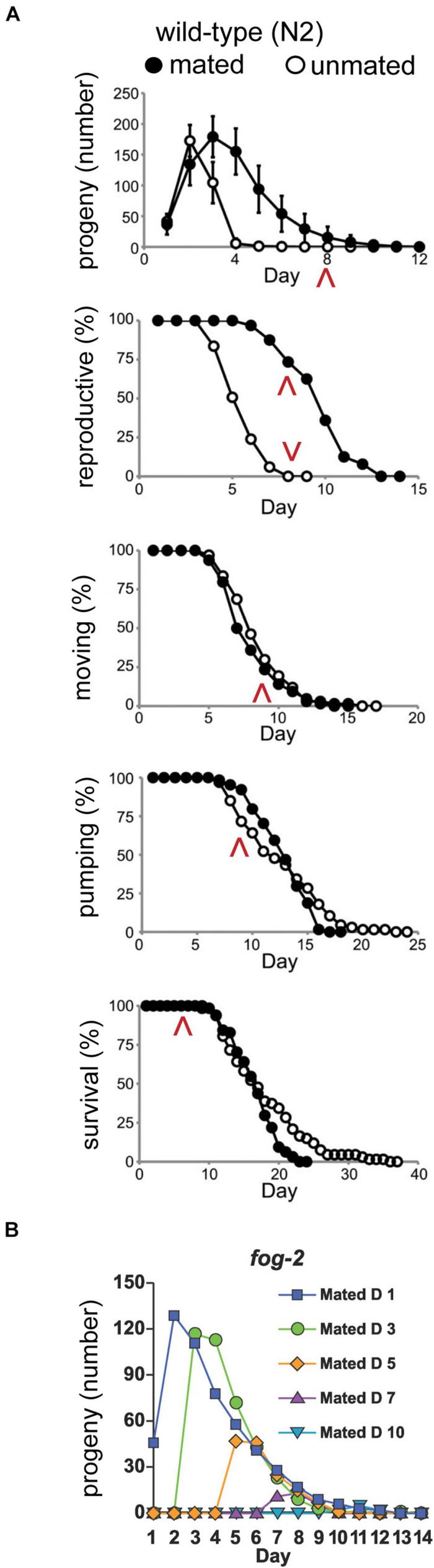
Somatic and reproductive aging are independent of progeny production. **(A)** Progeny production, reproductive span, body movement span, pharyngeal pumping span, and lifespan of mated and unmated wild-type hermaphrodites. The increase in amount and duration of progeny production caused by male mating does not accelerate somatic aging. Red arrowheads highlight day 8 in each span. Adapted from Pickett et al. (2013). **(B)** Feminized *fog-2(q71)* mutants were mated to wild-type males for 24–48 h at different ages. Progeny curves show that late progeny production and reproductive span after peak were independent of early progeny production. Adapted from Hughes et al. (2007).

The decline in self-fertile or mated reproductive capacity occurs relatively early in life, before age-related declines of somatic tissues required for neuromuscular activity and life support systems required for survival (Figures 1B,C, 2; Huang et al., 2004; Pickett et al., 2013). More specifically, mated progeny production declines from a peak of ∼150 progeny/day on adult day 2 to ∼40 progeny/day on adult day 5 and to ∼12 progeny/day on adult day 7, while day 7 adults are still feeding, moving, and alive (Kocsisova et al., 2019). This suggests that the age-related decline in reproductive function may not be primarily caused by a decline in the somatic functions that maintain survival.

To explicitly investigate the relationship between reproductive aging and somatic aging, investigators have used the approach of longitudinal analysis. By making serial measurements of age-related declines in a single individual, it is possible to determine if two age-related declines are correlated. Huang et al. (2004) showed that the total reproductive span is not correlated with measures of somatic aging such as movement span and lifespan in self-fertile hermaphrodites, consistent with the model that the reproductive span in these animals is controlled by the number of self-sperm and the rate of self-sperm utilization; the number of self-sperm is established in larvae and is not related to aging. By contrast, Pickett et al. (2013) showed that there is a positive correlation between the total reproductive span and measures of somatic aging such as lifespan in mated hermaphrodites. In other words, individual worms that produced progeny for an extended period also lived for an extended period. These results indicate that reproductive and somatic aging are controlled by similar processes, and highlight the importance of measuring reproductive aging in mated hermaphrodites.

Males can have multiple effects on hermaphrodites, including transfer of sperm, transfer of seminal fluid, and physical trauma. Thus, the results of mating experiments depend on the duration and intensity of mating, including the number of males and ratio of males to hermaphrodites. For studies of reproductive aging, the strategy is to minimize male exposure by using a small number of males for a brief duration. Hughes et al. (2007) showed that it is typically sufficient to expose hermaphrodites for 24–48 h with males to provide a lifetime supply of sperm. To ensure sperm are not depleted late in life, the investigator can monitor the sex ratio of progeny. Hermaphrodites that cease producing male progeny late in life are inferred to have run out of male sperm and can be censored from the analysis.

Early studies reported contradictory results of the effect of mating on hermaphrodites: Van Voorhies (1992) found minimal effects of mating on hermaphrodites, whereas Gems and Riddle (1996) reported dramatic effects with lifespans of mated hermaphrodites reduced ∼40% due to an increase in stress. Both groups used a 1.5 male to hermaphrodite ratio and retained the males for the duration of the lifespan. Pickett et al. (2013) systematically measured how brief, 24–48 h mating affects somatic aging. There was a minimal effect on somatic aging - the mean body movement span decreased 8%, the pharyngeal pumping span decreased 3%, and the mean lifespan decreased by 9% in mated compared to self-fertile hermaphrodites (Figure 2A). In contrast, Shi and Murphy (2014) described a shrinking phenotype for hermaphrodites mated for a period of 24 h and a more than 40% decrease in lifespan. Shi and Murphy (2014) concluded that seminal fluid transferred by males to hermaphrodites contributes to this effect. Other investigators concluded that males release pheromones that cause the decrease in the lifespan of mated hermaphrodites (Maures et al., 2014; Shi et al., 2017). In addition, the small molecule nacq#1 that is predominantly produced by males reduces the lifespan of hermaphrodites in picomolar concentrations (Ludewig et al., 2019). By contrast, self-sperm protects hermaphrodites against male toxicity (Booth et al., 2019; Shi et al., 2019). These studies highlight one of the challenges of analyzing female reproductive aging – the ability of females to produce fertilized progeny is the gold standard measure of reproduction and the typical endpoint in studies of reproductive aging. However, fertilized progeny require male sperm, and the acquision of male sperm inevitably involves confounding variables that must be considered and controlled.

## Using the Germ Line to Make Eggs Does not Accelerate Reproductive Aging

Many theories have suggested that aging is a use-dependent degenerative decline. In other words, the more a system is used, the more rapidly it will decline. The alternative model is that age-related degeneration is controlled by a “clock,” and degenerative change will occur whether or not the system is used. *C. elegans* reproductive aging provides a powerful system to test these concepts. In particular, by using self-sterile mutants, it is possible to fully control progeny production by controlling the time when hermaphrodites mate with males. Hughes et al. (2007) showed that the shape of the curve describing the decline in egg-laying was essentially the same for animals mated on day 1 or mated on later days (Figure 2B). Thus, making progeny early in life did not accelerate or delay reproductive decline, indicating that this process is regulated by a timing mechanism.

Theoretical work has also proposed that there is a tradeoff between reproduction and somatic maintenance, which appears to predict that high levels of early reproduction will cause an acceleration of somatic aging. Pickett et al. (2013) examined somatic aging in self-fertile and mated hermaphrodites. Although mated hermaphrodites produced many more progeny, there was very little difference between the declines of movement, pharynx pumping and survival probability (Figure 2A). Thus, progeny production does not shorten lifespan or accelerate somatic aging (Pickett et al., 2013). Furthermore, Pickett et al. (2013) performed longitudinal studies and discovered that in mated hermaphrodites, high levels of progeny are positively correlated with long lifespan. In other words, individuals that made the most progeny for the longest period also tended to live the longest. This provides further evidence that making eggs does not cause the somatic tissues to degenerate – if anything, it appears that making a large number of eggs is evidence of a robust individual that is likely to live an extended period.

## Age-Related Changes in Meiotic Recombination, Progeny Viability and Morphology

Mated hermaphrodites display a decrease in recombination frequencies, whereas older hermaphrodites display a higher rate of X-chromosome nondisjunction that results in the production of more males late in life (Rose and Baillie, 1979). In contrast to this early study, Lim et al. (2008) could not detect a difference in crossover frequency by analyzing chromosome III. However, they discovered that adult day 1 mated hermaphrodites exhibit different crossover positions compared to adult day 6/7 animals (Lim et al., 2008). A direct consequence of this age-related change in the location of crossovers is that early progeny clearly differ from late progeny.

**FIGURE 3:**
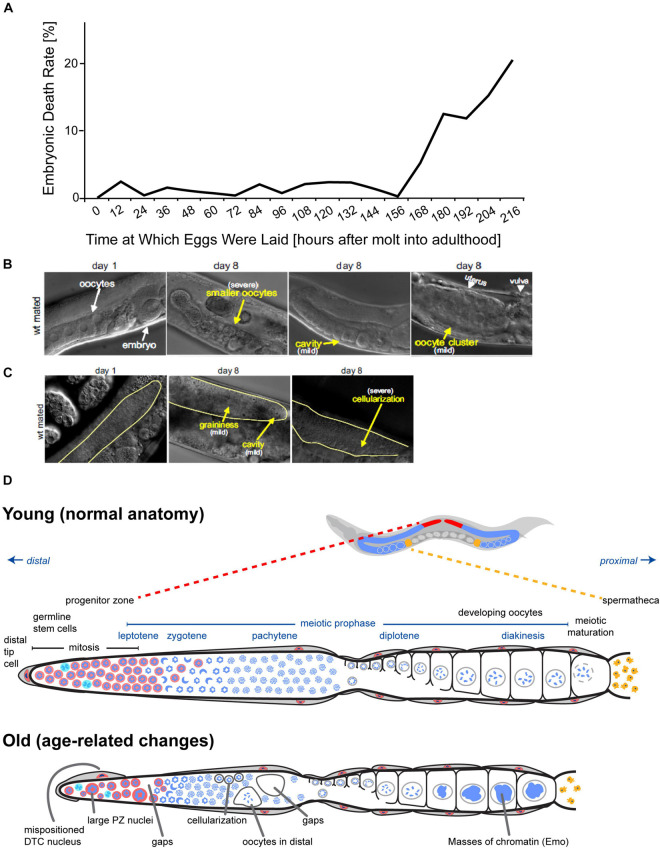
Age-related declines of oocyte and germline quality. **(A)** *fog-2(q71)* mutant animals that do not produce self-sperm were mated to males (start: 0 h), and the percentage of unhatched embryos was determined every 12 h. The embryonic death rate displays an age-related increase to ∼20% by day 9. Adapted from Andux and Ellis (2008). **(B)** Oocytes of adult day 8 mated wild-type hermaphrodites display morphological defects visible using differential interference contrast microscopy (DIC). Compared to adult day 1 mated wild-type hermaphrodites, oocytes in adult day 8 hermaphrodites can be smaller, clustered, or contain cavities (yellow arrows). Reprinted from Luo et al. (2010) with permission from Elsevier. **(C)** The distal germ line of adult day 8 mated wild-type hermaphrodites displays morphological defects visible using DIC microscopy. Compared to adult day 1 mated wild-type hermaphrodites, the distal germ line loses its smoothness in adult day 8 hermaphrodites and can appear grainy with cavities or exhibit severe cellularization (yellow arrows). The morphological defects are defined according Garigan et al. (2002). Reprinted from Luo et al. (2010) with permission from Elsevier. **(D)** Anatomy of *C. elegans* germ line in young versus old hermaphrodites. Top: *C. elegans* hermaphrodites have two U-shaped germ lines (red and blue). The spermatheca is shown in yellow, and the uterus with developing embryos is shown in dark gray. Middle: Diagram of one unfolded young *C. elegans* hermaphrodite germ line. Nuclear morphology can be visualized by staining DNA with DAPI (blue). The distal progenitor zone (red) contains mitotically cycling stem and progenitor cells. The distal tip cell (DTC) provides the GLP-1/Notch ligand to maintain the stem cell fate of these cells. As cells migrate away from the DTC, they exit the progenitor zone and enter meiotic prophase. Bottom: Diagram of one unfolded old *C. elegans* hermaphrodite germ line. Numerous age-related changes in the germ line have been reported. Several are illustrated here. Adapted from Kocsisova et al. (2019).

Self-fertile hermaphrodites produce embryos that have a high rate of viability; more than 99% of embryos laid will hatch (Andux and Ellis, 2008). Andux and Ellis (2008) also reported that lethality increased up to ∼20% in embryos that developed from stacked oocytes. Mated hermaphrodites have a larger reservoir of sperm, allowing them to continue producing progeny later in life. Embryos laid by mated and self-fertile hermaphrodites display a similar rate of lethality early in the reproductive span (adult day 1–4) that is lower than 1%. However, lethality among embryos that developed from non-stacked oocytes produced by older mothers was ∼3% (Andux and Ellis, 2008; Luo et al., 2010; Luo and Murphy, 2011). Andux and Ellis (2008) used sperm-defective *fog-2* “female” mutants to examine the effect of maternal age on embryonic viability. Until adult day ∼6.5, there is very little embryonic lethality. However, embryonic lethality displayed an age-related increase from adult days 6.5–9, culminating in about 20% lethality (Figure 3A). This suggests that maternal age has a negative effect on embryo viability, but it is important to consider the caveat that the *fog-2* mutation may affect germline aging beyond feminization. A practical limitation of these studies is the difficulty of obtaining large numbers of eggs produced by older hermaphrodites, since day 9 adults lay only a few eggs. Thus, it has been difficult to identify interventions that influence this phenotype.

Consistent with this functional analysis, morphological analysis of age-related changes in the germ line reveals smaller oocytes and oocyte clusters in wild-type hermaphrodites (Figures 3B,C; Luo et al., 2010). Hibshman et al. (2016) examined the effect of maternal age on hatched embryos. Younger mothers (day 0 of adulthood) produced larval progeny that were ∼205 μm in length; progeny length peaked at day 1 at ∼215 μm before declining slightly at day 2 (Hibshman et al., 2016). In contrast, Perez et al. (2017) reported that progeny from adult day 1 self-fertile hermaphrodites were significantly shorter than progeny from adult day 2 and 3 self-fertile hermaphrodites. The length of larvae from adult day 1 hermaphrodites was ∼250 μm at hatching and ∼860 μm 42 h post hatching; for adult day 2 hermaphrodites, larval length was ∼260 μm at hatching and ∼910 μm 42 h post hatching; for adult day 3 hermaphrodites, larval length was ∼270 μm at hatching and ∼940 μm 42 h post hatching (Perez et al., 2017). In addition, the brood size of adult day 1 progeny was ∼250 progeny, which is less than the brood size of adult day 2 or 3 progeny (∼320 progeny) (Perez et al., 2017).

## Matricidal Hatching

**FIGURE 4:**
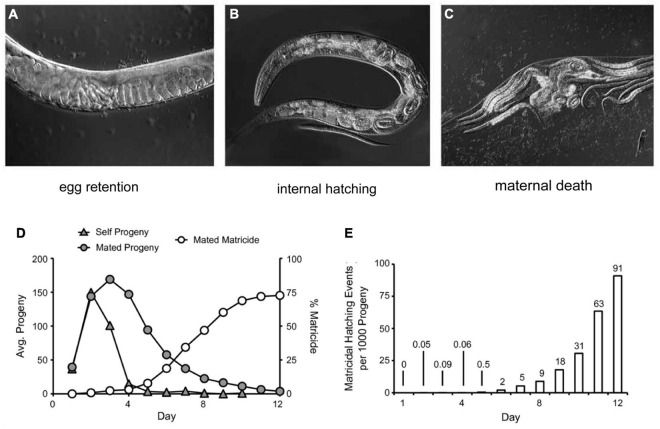
Matricidal hatching. Wild strain JU751 hermaphrodites exhibit nearly constitutive matricidal hatching. DIC microscopic micrographs depict the progression: **(A)** Eggs are retained in the uterus, showing a “stacking of embryos” phenotype. **(B)** Retained embryos will continue to develop and hatch inside the uterus. **(C)** Hatched larvae grow inside the hermaphrodite and feed on the maternal tissue. Finally, the hermaphrodite bursts and releases the progeny in dauer stage. **(A–C)** Adapted from Vigne et al. (2021). **(D)** The cumulative percentage of matricidal hatching in mated wild-type hermaphrodite in comparison to progeny production curves of unmated/self-fertile and mated wild-type hermaphrodites. Adapted from Pickett and Kornfeld (2013). **(E)** Incidence number of matricidal hatchings per 1,000 progeny laid by mated wild-type hermaphrodites. Until day 6, progeny production carries a small risk of matricidal hatching, but after day 6 the risk increases exponentially. Adapted from Pickett and Kornfeld (2013).

If a hermaphrodite stops laying eggs into the environment, then fertilized eggs inside the uterus continue developing and hatch internally after about 12.5 h. The hatched larvae consume the biomass of the hermaphrodite, resulting in maternal death (Figures 4A–C). The maturing larvae leave the maternal body, often as dauer stage larvae. This phenotype is known as matricidal hatching, endotokia matricida, facultative viviparity, or “bag-of-worms” (Luc et al., 1979; Pickett and Kornfeld, 2013). This phenotype is not unique to *C. elegans*; it can be observed in other Rhabditia species as well as non-nematode species (Johnigk and Ehlers, 1999; Luong et al., 1999). In *C. elegans*, matricidal hatching is caused by genetic defects, adaptive signals, or age-related degeneration of the egg-laying system: (1) Mutant animals with developmental defects in the egg-laying system cannot lay eggs and reproduce via matricidal hatching (Trent et al., 1983). This phenotype was the basis for genetic screens that identified a wide range of genes important for development of the vulva, vulval muscles, and HSN neurons that innervate the vulval muscles. In addition, these studies were also the basis for the discovery of many components of important highly-conserved regulatory pathways such as EGFR, Rb, and Notch. (2) Egg retention and matricidal hatching can also be triggered by food deprivation. Starving hermaphrodites stop laying eggs, probably as a strategy to ensure that fertilized eggs can survive (Chen and Caswell-Chen, 2004). The next generation, born as dauer larvae through matricidal hatching, is optimized to survive the deprivation conditions. (3) Hermaphrodites stop laying eggs as they get older due to age-related degeneration of the egg-laying apparatus (Pickett and Kornfeld, 2013). This age-related change is not caused by use-dependent damage. With increasing age, hermaphrodites lose the ability to respond to exogenous serotonin, which triggers egg-laying in young adults with a functional egg-laying system. Consequently, mated wild-type hermaphrodites with an extended reproductive span display an increase of 70% in the frequency of matricidal hatching compared to self-fertile hermaphrodites that cease progeny production several days earlier due to sperm depletion (Figures 4D,E; Pickett and Kornfeld, 2013). In a sense, there is a “race” between the age-related decline in egg production and the age-related decline in the system that deposits eggs into the environment. If the egg production system declines first, as is typical in self-fertile hermaphrodites, then animals display an extended post reproductive lifespan. However, if the age-related decline in the somatic systems that deposit eggs occurs first, as is typical in mated hermaphrodites, then death is caused by matricidal hatching and there is no post reproductive lifespan. Interestingly, the percentage of hermaphrodites that display matricidal hatching increases in strains with mutations that induce longevity and extend the reproductive span when mated to males: *daf-2*, *age-1*, *clk-1*, *isp-1*, *sma-2*, and *sma-3* (Pickett and Kornfeld, 2013). In some *daf-2(lf)* mutants, essentially 100% of mated hermaphrodites die from matricidal hatching.

Vigne et al. (2021) identified wild-type strains that display premature loss of sensitivity to exogenous serotonin. These strains develop normally and have the ability to lay eggs in the first hours of adulthood. However, in contrast to the standard wild-type strain N2, these strains start to retain eggs in the uterus on adult day 1 due to a lack of responsiveness to food availability. Thus, they exhibit near-constitutive matricidal hatching (Vigne et al., 2021).

## Age-Related Morphological, Molecular and Functional Deterioration of the Germ Line

**FIGURE 5:**
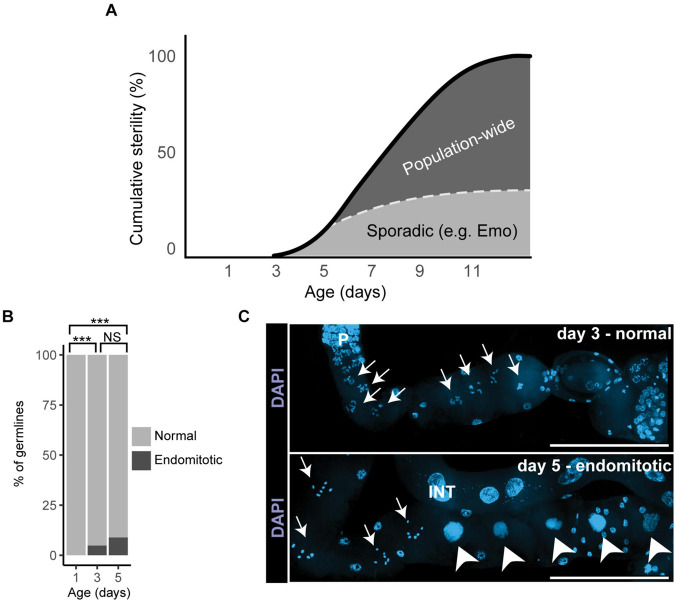
Sporadic degenerative changes in the aging germ line. **(A)** The graph illustrates how sporadic and population-wide changes contribute to cumulative age-related sterility in mated wild-type hermaphrodites. Endomitotic oocytes or shifted distal tip cells are sporadic degenerative changes and account for less than half of age-related sterility (the dotted line indicates a model of the relative contribution of sporadic defects). Declines in stem cell number and activity, PZ and germ line shrinking, as well as slower cell cycles are population-wide changes that appear to be the main drivers of reproductive aging. Adapted from Kocsisova et al. (2019). **(B)** Analysis of sporadic changes in day 1, 3, and 5 mated wild-type hermaphrodites: Percentage of dissected germ lines with (black) or without (gray) endomitotic oocytes (black). Adapted from Kocsisova et al. (2019). **(C)** Representative fluorescence micrographs of wild-type mated hermaphrodites stained with the DNA-dye DAPI. Oocytes of a day 3 adult show normal chromosomes in diakinesis (arrows in top panel) in contrast to endomitotic oocytes (arrowheads in lower panel) of a day 5 adult. P, pachytene; INT, intestine. Scale bars: 100 μm. Adapted from Kocsisova et al. (2019).

To ultimately understand mechanisms of reproductive aging, it is first necessary to have a detailed description of how the reproductive system functions in young animals and how it changes during aging. While these observational studies do not directly test the functional consequence of age-related changes, they are important for establishing hypotheses, and these phenotypes are the basis for evaluating interventions that influence reproductive aging. The germ line, somatic gonad, and more distant somatic tissues all contribute to reproduction, and it is important to understand how age-related changes in the reproductive tract and somatic tissues affect reproductive function. One important distinction when considering age-related changes is whether the change is observed at a low frequency in only a subset of animals or at a high frequency in all animals. Low frequency changes can be described as sporadic (Figure 5A), and this pattern suggests the cause may be stochastic events. By contrast, high frequency changes can be described as population-wide, and this pattern suggests the cause may be a genetic program or an environmental influence that is experienced by every animal.

**Low frequency, sporadic changes during reproductive aging**: If meiotic maturation and ovulation are miscoordinated, endomitotic oocytes occur, which then also disturb future successful coordination of these processes. Mutants with a high frequency of endomitotic oocytes (the Emo phenotype) have reduced fertility, indicating that this defect has functional consequences. Kocsisova et al. (2019) observed a small fraction of day 5 mated hermaphrodites that display endomitotic oocytes in the proximal gonad arm (Figures 3D, 5B,C). A longitudinal study demonstrated that this phenotype is positively correlated with reduced progeny production, indicating that in a subset of animals, endomitotic oocytes are a cause of age-related decline in fertility.

The distal tip cell (DTC) is a somatic cell positioned at the distal end of each gonad arm; the DTC provides the niche for germline stem cells and expresses Notch ligands that promote stem cell fate (Figure 1A). Kocsisova et al. (2019) reported that the DTC may mislocalize away from the distal tip of the gonad in day 5 mated adult hermaphrodites (Figure 3D). While severe defects in DTC position can cause obvious abnormalities in the gonad, severe defects are a low frequency, sporadic event that is not likely to explain the population-wide declines in egg production.

**High frequency, population-wide changes during reproductive aging**: The structure of the gonad undergoes visible deterioration as the animal ages (Figures 3C,D). It is challenging to quantify these morphological changes, and several investigators have established approaches using high magnification DIC optics. In a pioneering study, Garigan et al. (2002) defined five morphological categories based on the degree of disorganization, allowing a numeric score for each individual. Within the gonad, changes in the process of oocyte maturation and ovulation occur with age. In unmated hermaphrodites, sperm depletion results in an arrest of oocyte maturation, resulting in unfertilized oocytes “stacking” in the proximal gonad (Luo et al., 2010; Hughes et al., 2011). Hermaphrodites older than 8 days display a disorganization of the distal germ line with cavities, contraction into clumps, vacuoles, debris, graininess and cellularization (Figures 3C,D; Luo et al., 2010; Hughes et al., 2011). Hughes et al. (2011) established approaches to quantify deterioration and showed that mutations that delay reproductive aging can also delay age-related changes in morphology. While this type of evidence is suggestive that the morphological changes cause the functional decline, this correlation does not rigorously establish cause.

**FIGURE 6:**
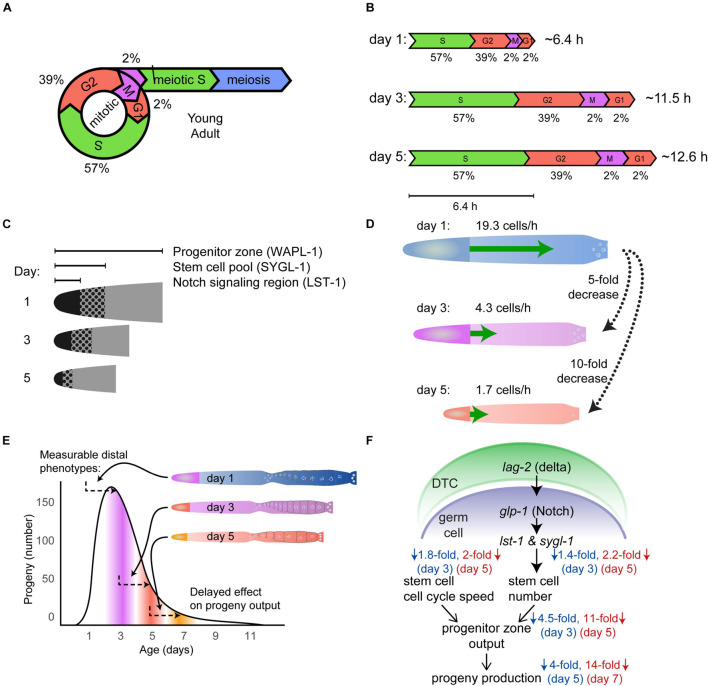
Population-wide, age-related degenerative changes in the germ line. **(A)** Schematic of the mitotic cell cycle and meiotic entry of young adult wild-type germ cells, including duration of time in each phase expressed as a percent (Fox et al., 2011). Adapted from Kocsisova et al. (2019). **(B)** The duration of the cell cycle increases with age in mated wild-type hermaphrodites. The scale diagram summarizes the duration of different phases of the cell cycle in day 3 and 5 animals compared to day 1 animals. The values below indicate the percentage of time in each phase, whereas the values on the right indicate the total cell cycle duration time. Adapted from Kocsisova et al. (2019). **(C)** The schematic shows that the size of the Notch signaling region, stem cell pool, and the progenitor zone (PZ) shrinks with age. Notch signaling was visualized by LST-1 labeling, the stem cell pool was visualized by SYGL-1 labeling, and the progenitor zone was labeled with WAPL-1 antibodies in day 1, 3, and 5 germ lines. Adapted from Kocsisova et al. (2019). **(D)** The schematic displays the age-related decrease in the rate of meiotic entry (green arrow) in the germ line of mated wild-type hermaphrodites. Day 5 and 3 germ lines show a 10-fold and 5-fold decrease, respectively, compared to day 1 germ lines. Adapted from Kocsisova et al. (2019). **(E)** The schematic shows that changes in the distal germ line correlate with changes in the progeny production curve about 2 days later (arrows). The size of the germ line and the progenitor zone (PZ) decreases before the progeny production declines. The PZ cells need two or more days to become oocytes and be laid as eggs. Therefore, age-related changes such as the shrinking of the germ line and PZ affect progeny production with a delay. Adapted from Kocsisova et al. (2019). **(F)** Summary and model of reproductive aging in *C. elegans*. Adapted from Kocsisova et al. (2019).

To gain insight beyond the morphological changes observable with DIC optics, investigators began using sophisticated assays developed to measure germ line function in young animals, including antibody staining to identify regions of the germ line and phases of the cell cycle. Furthermore, by using the nucleotide analog EdU that is incorporated into newly synthesized DNA, it is possible to observe the dynamics of DNA replication, duration of the cell cycle, and the rate of germ cell progression through the gonad. In young adult hermaphrodites, the germline stem cells of the progenitor zone (PZ) take 6.5–8 h to go through one complete round of the cell cycle (Fox et al., 2011). The M-phase index (percent of cells in M-phase at a moment in time) decreases with age in unmated hermaphrodites, suggesting an arrest in the germ cell cycle (Narbonne et al., 2015; Qin and Hubbard, 2015); mating to males delays this decline (Narbonne et al., 2015). Kocsisova et al. (2019) reported an age-related increase in the time necessary to complete the cell cycle, increasing to ∼12 h in day 3 and 5 adult mated hermaphrodites (Figures 6A,B). Notably, this increase in cell cycle duration was measured on adult day 3, about 2 days before there is a substantial decline in progeny production, indicating that declines in the distal germ line may be the first changes during reproductive aging.

In the distal most end of the germ line near the DTC, germ cells undergo mitotic division in the PZ. In young adult self-fertile hermaphrodites, there are ∼200–250 mitotically dividing cells in this region (there is some variation in this value determined by different laboratories) (Killian and Hubbard, 2005; Crittenden et al., 2006; Luo et al., 2010; Narbonne et al., 2015; Qin and Hubbard, 2015). Some laboratories observed changes in the number of cells in the PZ in older hermaphrodites: Luo et al. (2010) reported a decrease to ∼150 cells at day 6; Killian and Hubbard (2005) as well as Qin and Hubbard (2015) observed ∼150 cells at day 3, ∼100 cells at day 6, and ∼50 cells at day 12; Narbonne et al. (2015) observed ∼80 cells at day 7. Variation in the PZ cell number has been observed between self-fertile and mated hermaphrodites – several laboratories reported a more rapid decline in PZ cell number in mated animals, suggesting that the PZ may become more rapidly depleted in mated animals (Figure 6C; Shi and Murphy, 2014; Narbonne et al., 2015; Qin and Hubbard, 2015; Kocsisova et al., 2019). Apoptosis plays an important functional role in reducing the number of germ cells in late pachytene during early adulthood, and a possible explanation for the age-related reduction in stem cell number is “apoptotic run on” (de la Guardia et al., 2016). The discovery of age-related increases in apoptotic germ cells and the ratio of apoptotic cells/germ cells supports the hypothesis that excess apoptosis causes the age-related reduction in stem cell number, which may be an example of an unproductive run-on program (de la Guardia et al., 2016).

Moving proximally down the germ line, in the meiotic entry region, germ cells begin entering meiotic prophase. In young adults, the process of meiotic prophase progression and oogenesis takes ∼2 days, and has been observed to slow in day 1.5 adults (Jaramillo-Lambert et al., 2007). Schisa et al. (2001) reported an increase in perinuclear staining of RNA-rich “P granules” in the germ line of old adult hermaphrodites compared to young hermaphrodites. Kocsisova et al. (2019) used EdU labeling to measure the movement rate of cells exiting the PZ. The rate of movement declined from ∼19.3 cells/hours on adult day 1 to ∼4.3 cells/hours on adult day 3 to ∼1.7 cells/hour on adult day 5 (Figure 6D). This is a remarkable and early change that strongly points to the distal germ line as the origin of reproductive aging (Figure 6E).

The DTC signals the germ line to specify stem cell fate via the GLP-1 Notch pathway. As germ cells move proximally away from the distal tip of the gonad, they lose Notch signaling and transition from mitotic cell cycling into meiotic S-phase (Fox and Schedl, 2015). *lst-1* and *sygl-1*, two germline transcriptional targets of GLP-1 signaling, are known to be redundantly necessary and individually sufficient for promoting stem cell fate (Kershner et al., 2014; Lee et al., 2016; Shin et al., 2017). Observing the number of cells that express these proteins provides information about the extent of Notch signaling. The number of cells expressing LST-1 and SYGL-1 in day 1 adult mated hermaphrodites is ∼38 for LST-1 and ∼81 for SYGL-1; this number is reduced by half by day 5 (Figures 6C,F; Shin et al., 2017; Kocsisova et al., 2019). This is an example of a molecular read out – the pattern and level of protein expression - that displays an age-related change and indicates that there is a progressive reduction of the Notch-mediated signaling system that maintains germline stem cells.

## Pharmacological Compounds That Influence Reproductive Aging

**FIGURE 7:**
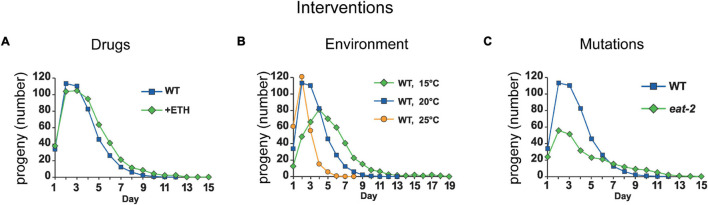
Environmental, genetic, and pharmacological factors can delay reproductive aging. Representative interventions that prolong the reproductive span and increase late progeny production in short (24 h) mated *C. elegans* hermaphrodites. **(A)** Ethosuximide-treatment increases mid-life and late progeny production and prolongs the reproductive span in mated wild-type *C. elegans*. Adapted from Hughes et al. (2007). **(B)** Increasing the culture temperature from 20°C (blue curve) to 25°C (yellow curve) decreases late progeny production and reduces the reproductive span after peak in mated wild-type *C. elegans*. By contrast, reducing the culture temperature to 15°C (green curve) decreases early progeny production, increases late progeny production, increases reproductive span to peak, and extends the reproductive span after peak in mated wild-type *C. elegans*. Adapted from Hughes et al. (2007). **(C)** Mated *C. elegans* hermaphrodites with a mutation in *eat-2* (green) display decreased early progeny production, decreased peak progeny number, increased late progeny production, and an extended total reproductive span compared to wild type. Adapted from Hughes et al. (2007).

To move beyond descriptions of age-related changes to an understanding of mechanism, it is necessary to identify interventions that can influence reproductive aging. *C. elegans* is a powerful model to screen for such interventions, and it has been a leading system for innovative discovery in this area. Here we consider three categories of interventions that have been demonstrated to delay reproductive aging: drugs, single gene mutations, and environmental factors (Figure 7, Tables 1, 2 and Supplementary Table 1). We focus on delayed reproductive aging rather than accelerated reproductive aging because there are many ways to reduce reproduction, and only a small number of these are likely to be relevant to aging. By contrast, it is relatively rare to find interventions that extend reproduction, and these are more likely to modulate the aging process. An important issue is what assays or measurements constitute strong evidence for delayed reproductive aging? As illustrated in Figure 1E, the most stringent and reliable are (1) increases in the total reproductive span, which is conceptually similar to extended lifespan for studies of somatic aging, and (2) increases in the number of viable progeny produced late in life, which is conceptually similar to extended healthspan for studies of somatic aging. *C. elegans* has long been used as a model to examine the effects of pharmacological compounds on aging. To date, many compounds have been shown to extend nematode lifespan (Lucanic et al., 2013, 2017); however, only a few of these compounds have been shown to delay reproductive aging (Table 1).

**TABLE 1:** Compounds that modulate reproductive aging in *Caenorhabditis elegans*.

Intervention^1^	Longitudinal experiments^2^/ Male mated^3^	Reproductive span to peak (days)^4^	Reproductive span after peak (days)^5^	Total reproductive span (days)^6^	Peak progeny number^7^	Total progeny number^8^	Matricidal hatching^9^	References
H_2_S by means of FW1256 (500 μM)	yes/no	↑	↓	↓	↑	+/−	ND	Ng et al., 2020
Ethosuximide (4 mg/ml)	yes/yes day1	*+/−*	↑	↑	+/−	+/−	reduced	Hughes et al., 2007
Trehalose (5 mM)	yes/no	+/−	↑	↑	+/−	+/−	ND	Honda et al., 2010
Resveratrol (50 μM)	yes/no	↑	+/−	↑	↓	+/−	ND	Gruber et al., 2007
DMSO (0.8% v/v)	yes/no	↑	↓	↑	↓	↓	ND	Frankowski et al., 2013
DMF (0.75% v/v)	yes/no	↑	↑	↑	↓	↓	ND	Frankowski et al., 2013
Vitamin E (400, 800 μg/mL)	yes/no	↑	↑	↑	↓	↓	ND	Zuckerman and Geist, 1983; Harrington and Harley, 1988
Folic acid (25 μM)	yes/no	+/−	↑	↑	↓	↓	ND	Rathor et al., 2015
Metformin (10, 50 μM)	yes/no	↑	↑	↑	↓	↓	ND	Onken and Driscoll, 2010; Cabreiro et al., 2013

*^1^Intervention: pharmacological compounds (concentration in medium).*

*^2^Longitudinal experiment: the same individual animal is observed at sequential time points. yes: measured in a longitudinal experiment, no: measured in a cross-sectional experiment.*

*^3^Male mated: yes – hermaphrodites were exposed to males, with adult day of exposure specified, no: self-fertile hermaphrodites.*

*^4^Reproductive span to peak: time from adult day 0 until day of peak egg-laying (Figure 1).*

*^5^Reproductive span after peak: time from day of peak egg-laying until last day of egg-laying (Figure 1).*

*^6^Total reproductive span: time from adult day 0 until last day of egg-laying (Figure 1).*

*^7^Peak progeny number: Number of eggs laid on day of peak progeny production (Figure 1).*

*^8^Total progeny number: Total number of eggs laid (Figure 1).*

*^9^Matricidal hatching: If mature hermaphrodites cannot deposit eggs into the environment, then eggs hatch inside the hermaphrodite, and larvae feed on the parent resulting in death.*

*^4–9^ ↑: increase; ↓: decrease; +/−: no change; reduced: lower frequency; ND: not determined.*

**TABLE 2:** Environmental interventions that modulate reproductive aging in *Caenorhabditis elegans*.

Intervention^1^		Longitudinal experiments^2^/ Male mated^3^	Reproductive span to peak (days)^4^	Reproductive span after peak (days)^5^	Total reproductive span (days)^6^	Peak progeny number^7^	Total progeny number^8^	Matricidal hatching^9^	References
Temperature	WT 15°C	yes/yes day1	↑	ND	↑	↓	+/−	ND	Hughes et al., 2007
	WT 25°C	yes/yes day1	+/−	ND	↓	+/−	↓	ND	Hughes et al., 2007
Bacterial/food source in comparison to *E. coli* OP50	Staphylococcus epidermidis	yes/no	↑	↑	↑	ND	+/−	ND	Madhu et al., 2019
	*E. coli K-12* HT115”	yes/no	+/−	↓	↓	↑	+/−	ND	Stuhr and Curran, 2020
	*E. coli* B&*K-12* HB101”	yes/no	+/−	↓	↓	↑	+/−	ND	Stuhr and Curran, 2020
	*Sphingomonas aquatilis* “Yellow”	yes/no	+/−	↓	↓	+/−	↓	ND	Stuhr and Curran, 2020
	*Xanthomonas citri* “Orange”	yes/no	+/−	↓	↓	↑	+/−	ND	Stuhr and Curran, 2020
	*Methylobacterium braciatum* “Red”	yes/both	+/−	↓	↓	+/−	↓	ND	Stuhr and Curran, 2020
Fungi	Yeast *Candida albicans*	yes/no	+/−	+	+	↓	+/−	ND	Feistel et al., 2019

*^1^Intervention: Temperature or bacterial food source.*

*^2^Longitudinal experiment: the same individual animal is observed at sequential time points. yes: measured in a longitudinal experiment, no: measured in a cross-sectional experiment.*

*^3^Male mated: yes – hermaphrodites were exposed to males, with adult day of exposure specified, no: self-fertile hermaphrodites.*

*^4^Reproductive span to peak: time from adult day 0 until day of peak egg-laying (Figure 1).*

*^5^Reproductive span after peak: time from day of peak egg-laying until last day of egg-laying (Figure 1).*

*^6^Total reproductive span: time from adult day 0 until last day of egg-laying (Figure 1).*

*^7^Peak progeny number: Number of eggs laid on day of peak progeny production (Figure 1).*

*^8^Total progeny number: Total number of eggs laid (Figure 1).*

*^9^Matricidal hatching: If mature hermaphrodites cannot deposit eggs into the environment, then eggs hatch inside the hermaphrodite, and larvae feed on the parent resulting in death.*

*^4–9^ ↑: increase; ↓: decrease; +/−: no change; ND: not determined.*

**Ethosuximide** is an anticonvulsant medication commonly used to treat seizures in humans. Evason et al. (2005) discovered that exposure to ethosuximide at 4 mg/mL in the medium resulted in a 17% increase in mean adult lifespan, as well as an increase in the amount of time that animals displayed fast body movement and pharyngeal pumping, indicating that ethosuximide also delays age-related physiological decline (Evason et al., 2005). Ethosuximide treatment does not affect self-fertile reproductive span, but mated hermaphrodites displayed an increase in the reproductive span of 12% (Evason et al., 2005; Hughes et al., 2007). Notably, ethosuximide did not reduce progeny production early in life (Figure 7A and Table 1). In addition, ethosuximide protects against matricidal hatching (Scharf et al., 2016). Ethosuximide is likely to function by inhibiting the activity of amphid neurons (Collins et al., 2008). These results suggest that it is possible to increase the reproductive span without affecting early reproduction, and that sensory perception by amphid neurons accelerates somatic and reproductive aging.

**Trehalose** is a dissacharide used as a human food additive. Honda et al. (2010) observed that trehalose at a concentration of 5mM in the medium extended mean lifespan by ∼30%. In addition, trehalose treatment extended the maximum reproductive span of wild-type (N2) self-fertile hermaphrodites: untreated animals stopped producing progeny at day 11 of adulthood, while trehalose treated animals continued producing progeny to day 15 (Table 1; Honda et al., 2010). Furthermore, trehalose treatment increased the mean reproductive span from 7.9 days to 11.0 days (Honda et al., 2010).

**Resveratrol** is a stilbenoid, naturally occurring phenolic compound found in foods such as grapes/red wine that has been demonstrated to affect aging in several organisms (Akinwumi, 2018). In *C. elegans*, resveratrol treatment at 50 μM in the medium extended mean lifespan by 64% and maximum lifespan by 30% (Gruber et al., 2007). Resveratrol treatment reduced the number of progeny produced early in life (defined as day 0–4 of adulthood) while increasing the number of progeny produced later in life (day 5–9); the total number of progeny produced was unaffected (Table 1; Gruber et al., 2007).

**Dimethyl sulfoxide (DMSO)** and dimethyl formamide (DMF) are amphipathic solvents commonly used in the chemical and biological sciences. Due to their ability to dissolve both polar and nonpolar compounds, they are often used to deliver pharmacological compounds in drug screens. Interestingly, treating nematodes with these solvents alone was reported to extend lifespan by 20 and 50% for DMSO (0.8% v/v) and DMF (0.75% v/v), respectively (Frankowski et al., 2013). These compounds also significantly reduced brood size and delayed the onset of egg-laying, while also extending the egg-laying period (Table 1; Frankowski et al., 2013).

**Vitamin E** has long been known to extend lifespan in many animals (Zuckerman and Geist, 1983). In *C. elegans*, vitamin E administration at 400-800μg/mL in the medium increased mean lifespan by 17–23% (Harrington and Harley, 1988). Vitamin E treatment reduces total progeny production (Zuckerman and Geist, 1983; Harrington and Harley, 1988); however, it also increases the mean days of reproduction (Table 1; Harrington and Harley, 1988).

**Folic acid** is a naturally occurring compound found in leafy vegetables, beans, and fruits. Treatment with folic acid at 25μM in the medium extends mean worm lifespan by 26% (Rathor et al., 2015). The final day of progeny production increases from day 10 of adulthood to day 12, and total progeny production was reduced (Table 1; Rathor et al., 2015).

**Metformin** is a drug commonly used to treat type-2 diabetes in humans; it extends lifespan in non-diabetic mice, as well as reducing all-cause mortality in non-diabetic humans (Scarpello, 2003; Anisimov et al., 2011). Onken and Driscoll (2010) observed that metformin treatment at 10 or 50 μM in the medium increased median lifespan by 40%. Metformin treatment reduced the number of eggs laid early in the reproductive period, while also extending the total reproductive period by 1 day (Table 1; Onken and Driscoll, 2010). Notably, this phenotype is similar to the effects caused by dietary restriction (DR). Cabreiro et al. (2013) also analyzed metformin and observed that lifespan extension depends on the bacterial food source; they concluded that metformin extended worm lifespan by interrupting bacterial methionine and folate metabolism, inducing a DR phenotype in worms via methionine restriction, rather than working directly on *C. elegans* metabolism (Cabreiro et al., 2013). These findings are an important cautionary tale regarding the complexity of interpreting drug studies in *C. elegans* when worms are feeding on live bacteria, and the bacteria may also be influenced by the drug.

3-dihydro-2-phenyl-sulfanylenebenzo[d] [1,3,2]-oxa-zaphosphole or FW1256, is an **H_2_S donor compound** initially prepared by Feng et al. (2015). Treatment with 500 μM FW1256 in the medium extends lifespan and health span of wild-type hermaphrodites and also shifts the peak of progeny production from day 5 to day 7 (Ng et al., 2020). The total number of progeny on day 4 and 5 are decreased, while the number of eggs on day 6–9 are increased, shifting the progeny curve to the right. The total number of eggs per hermaphrodite is slightly (not significant) increased. Even taking a slight developmental delay of a little less than one day (about 20 h) into consideration, this treatment still delays reproductive aging (Table 1).

An important observation in aging biology is that mutations in genes involved in distinct aging regulatory pathways can additively influence lifespan. In a similar fashion, pharmacological inhibition of targets that affect disparate longevity pathways can sometimes have additive effects on aging. Admasu et al. (2018) discovered that drugs targeting different pathways can further extend lifespan compared to animals treated with single drugs alone; notably, the combination of rapamycin (an mTORC1 inhibitor), allantoin (a DR mimetic), and either psora-4 (a K-channel blocker) or rifampicin (a JNK inhibitor) can extend the reproductive span of adult hermaphrodites while not reducing overall brood size (Admasu et al., 2018).

## Genetic Pathways That Influence Reproductive Aging

### Insulin/IGF-1 Signaling Pathway

**FIGURE 8:**
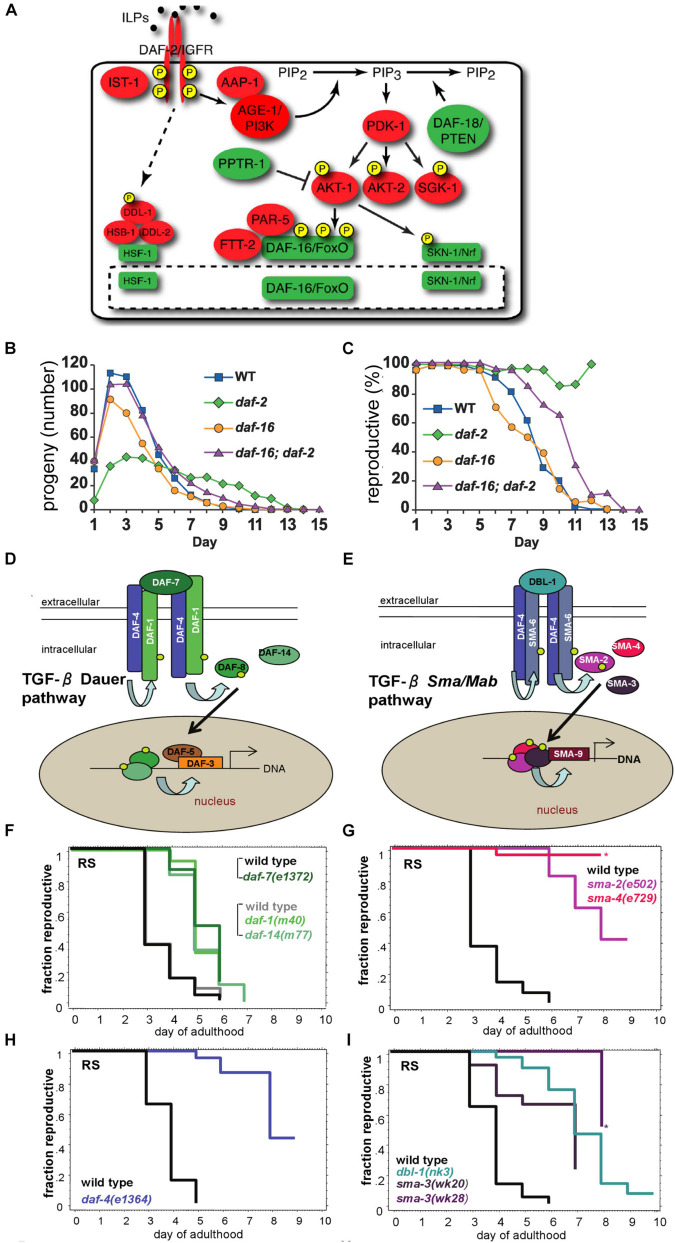
Mutations in the TGF-β and the Insulin/IGF-1 signaling pathways increase reproductive capacity. **(A)** The schematic depicts the Insulin/IGF-1 signaling pathway. Adapted from Murphy and Hu (2013). **(B)** Mated hermaphrodites with a mutation in the *daf-2* gene (green) display reduced peak progeny number, increased late progeny production, and an extended total reproductive span. By contrast, mated *daf-16* mutant hermaphrodites display a reduced peak progeny number, a reduced total progeny number, and a reduced total reproductive span. Adapted from Hughes et al. (2007). **(C)** Mated *daf-2* mutants show a higher percentage of live hermaphrodites producing progeny late in life compared to mated *daf-2;daf-16* double mutants, mated *daf-16* mutants or mated wild-type animals. Adapted from Hughes et al. (2007). **(D,E)** The schematics depict the TGF-β Dauer pathway and the TGF-β *Sma/Mab* pathway. Adapted from Luo et al. (2009). **(F–I)** *C. elegans* hermaphrodites with mutations in the TGF-β Dauer pathway **(F,H)** or TGF-β *Sma/Mab* **(G,I)** display an extended reproductive span compared to wild type. Adapted from Luo et al. (2009).

The **Insulin/IGF-1 signaling pathway** (Figure 8A) is the most prominent pathway in *C. elegans* aging research, as the first mutations discovered to extend lifespan affected genes encoding Insulin/IGF-1 signaling pathway components (Friedman and Johnson, 1988; Kenyon et al., 1993). The heart of this pathway is DAF-2, the only insulin-like receptor in *C. elegans*. In contrast, *C. elegans* can express about 40 insulin-like molecules that could in principle bind DAF-2; however, the peptides that bind DAF-2 during different processes is an active area of research and much remains unknown. Activated DAF-2 initiates a kinase cascade that results in phosphorylation of DAF-16, a FoxO transcription factor. Phosphorylated DAF-16 does not accumulate in the nucleus, and therefore cannot regulate transcription of its target genes. The Insulin/IGF-1 pathway is conserved in all eukaryotes. It connects the control of growth, reproduction, metabolism, and aging to nutrient status (Murphy and Hu, 2013).

Loss-of-function mutations in the *daf-2* gene result in lifespan extensions, because DAF-16 accumulates in the nucleus and constitutively regulates target genes that promote longevity. In contrast, loss-of-function mutations in the *daf-16* gene shorten lifespan. The Insulin/IGF-1 signaling pathway also impacts reproductive aging (Figures 8B,C and Supplementary Table 1). Loss-of-function mutations in the *daf-2* and *age-1* genes extend the reproductive span in mated *C. elegans* (Hughes et al., 2007; Luo et al., 2009); these mutant hermaphrodites display decreased early reproduction, increased late reproduction, and an increase in the percentage of reproductively active hermaphrodites (Figures 8B,C and Supplementary Table 1). Consistent with these observations from candidate gene approaches, *daf-2* and other insulin/IGF-1 signaling pathway genes were identified in an unbiased screen for reproductive span extension (Wang et al., 2014). The reproductive aging delay in *daf-2* mutants is dependent on *daf-16*, similar to the lifespan extension (Hughes et al., 2007; Luo et al., 2009). Morphological analysis revealed that the germ line in *daf-2* mutants remains intact for longer and displays delayed age-related deterioration (Garigan et al., 2002; Luo et al., 2010). By contrast, hermaphrodites with *daf-16* mutations display an accelerated age-related deterioration of the germ line (Garigan et al., 2002). Qin and Hubbard (2015) showed that the age-related depletion of the progenitor zone is *daf-2* dependent. *daf-2* mutants displayed a longer period of maintenance of the progenitor zone that is dependent on *daf-16*, but in a manner that is largely non-autonomous and has a different anatomical focus from lifespan extension (Qin and Hubbard, 2015). Loss-of-function mutations in *daf-16* reduce the number of reproductively active hermaphrodites prematurely, reduce total and peak progeny number, and cause a shorter reproductive span (Figures 8B,C and Supplementary Table 1; Hughes et al., 2011).

### TGF-β Dauer Pathway and TGF-β Sma/Mab Pathway

In *C. elegans*, two partially related Transforming Growth Factor-β (TGF-β) pathways, the TGF-β (*daf-7)* dauer and the TGF-β (*dbl-1*) Sma/Mab pathways (Savage-Dunn, 2005; Gumienny and Savage-Dunn, 2013), have been implicated in aging (Shaw et al., 2007; Kaplan et al., 2015; Tissenbaum, 2015; Tominaga and Suzuki, 2019). Both pathways act through *daf-4* (Figures 8D,E) (Luo et al., 2009). These pathways are highly conserved at the molecular and functional level in many species, including *Drosophila* and humans (Gumienny and Savage-Dunn, 2013; Monsivais et al., 2017). Not only are these pathways associated with somatic aging, they are also associated with reproductive aging. In particular, loss-of-function caused by chromosomal mutations or RNA interference (RNAi) of genes in the TGF-β Sma/Mab pathways, including the type-II receptor gene *daf-4*, R-Smad genes *sma-2* and *-3*, Co-Smad genes *sma-4* and *sma-9* (*C. elegans* homolog of Schnurri (Foehr et al., 2006)), and the ligand gene *dbl-1*, all significantly affect the fraction of animals that are reproductive at an older age and produce progeny curves of varying shapes (Figures 8F–I and Supplementary Table 1). Although genes in the TGF-β dauer pathway extend the reproductive span (e.g., the ligand *daf-7* and *crh-1*, a *C. elegans* homolog of mammalian CREB (Templeman et al., 2020), antagonistic Co-Smad *daf-3*, type-I receptor *daf-1* and R-Smad *daf-14*), their effects are less robust compared to the TGF-β Sma/Mab pathway (Luo et al., 2009, 2010; Gumienny and Savage-Dunn, 2013). The type-II receptor gene *daf-4*, which is also involved in the TGF- β dauer pathway, causes a greater effect on reproduction (Figure 8H; Luo et al., 2009). The TGF-β Sma/Mab pathway was found to delay reproductive aging by maintaining oocyte and germline quality cell non-autonomously by acting in the hypodermis (Luo et al., 2010; Templeman et al., 2020). A genome-wide RNAi screen by Wang et al. (2014) for extended reproduction identified many genes associated with these two pathways, in addition to several genes related to sodium homeostasis (*nhx-2* and *sgk-1*) (Supplementary Table 1).

### Dietary Restriction

Dietary Restriction (DR) has long been established to extend lifespan in many species of animals, including mice, fruit flies, and nematodes (McCay et al., 1935; Kapahi et al., 2017; Krittika and Yadav, 2019). This intervention has been of particular interest in the field of aging due in part to its highly conserved nature.

Several genetic mutations have been identified in *C. elegans* that can lead to DR. Mutations in the *eat-2* and *phm-2* genes extend mean adult lifespan, reduce adult body size, reduce total brood size, and extend the reproductive period (Figure 7C and Supplementary Table 1; Lakowski and Hekimi, 1998; Hughes et al., 2007; Kumar et al., 2019). These mutations alter the structure or function of the pharynx, thereby reducing the pumping rate or altering the pumping motion (Lakowski and Hekimi, 1998; Hughes et al., 2011; Kumar et al., 2019). Indeed, the *phm-2(am117)* mutation was first identified in a screen for delayed reproductive aging, prior to being molecularly identified (Hughes et al., 2011). However, recent studies by Kumar et al. (2019) indicate that the interpretation of these mutations is more complicated than anticipated. Coordinated pharyngeal pumping brings bacterial food into the organism and also grinds the bacteria so that they cannot colonize the intestine. Mutations in *eat-2* and *phm-2* cause pharyngeal defects that impair bacterial grinding, which allows live bacteria to enter and colonize the intestine of young adults. Thus, these mutants are immunocompromised. Because the standard bacterial food source, *E. coli* OP50, is mildly pathogenic, intestinal colonization provokes the innate immune response, which includes bacterial avoidance behavior. The bacterial avoidance behavior appears to cause dietary restriction in these mutants rather than an inability to ingest enough bacteria. Because these mutations lead to bacterial pathogenesis, an innate immune response, and bacterial avoidance behavior that leads to DR, any or all of these problems might contribute to the effect on reproductive aging.

Several non-genetic methods have been used to induce DR in worms, including dilution of the bacterial food source or interruption of food availability via intermittent fasting (Kaeberlein et al., 2006; Lee et al., 2006; Bishop and Guarente, 2007; Panowski et al., 2007; Chen et al., 2009; Greer and Brunet, 2009). Bishop and Guarente (2007) demonstrated that hermaphrodites grown in liquid medium with restricted bacterial concentration display reduced brood size and an extended reproductive period, ceasing reproduction on adult day 14 compared to day 8 in ad-libitum fed animals (Bishop and Guarente, 2007). Chen et al. (2009) demonstrated that a reduction in bacterial concentration was correlated with reduced fecundity (Chen et al., 2009). A change from 1.0 × 10^12^ cfu/mL to 1.0 × 10^8^ cfu/mL resulted in a reduction in brood size from ∼200 to ∼50, but with no observable change in the length of the reproductive period. Houthoofd et al. (2002) observed that culture in axenic liquid media reduced brood size and extended the reproductive period. Szewczyk et al. (2006) used the chemically defined growth medium CeMM and observed a reduction in brood size from ∼240 to ∼81, as well as an extension of the reproductive period from adult days 2–4 to days 7–14.

It has long been theorized that the effect of DR on lifespan is the result of a trade-off in caloric investment between reproduction and maintenance of somatic tissues (Holliday, 1989). However, studies in nematodes have shown that reduction in reproductive capacity is not necessary for lifespan extension by DR (Kaeberlein et al., 2006). Additionally, many studies have shown that various methods of DR still extend lifespan in nematodes treated with 5-fluorodeoxyuridine (FUDR, an inhibitor of nematode reproduction), suggesting that lifespan extension is independent of reproduction with respect to DR (Kaeberlein et al., 2006; Panowski et al., 2007; Greer and Brunet, 2009). We discuss this issue further in the section below regarding reproductive aging and evolutionary theories of aging.

### GABAergic Signaling

Cermak et al. (2020) recently reported the importance of GABAergic signaling for dopamine-induced egg-laying. The GABAergic-signaling-defective *unc-25(lf)* mutations delay egg-laying by one day. Compared to wild-type worms, which display an egg-laying peak on adult day 2, *unc-25* mutants display a peak on adult day 3; *unc-25* mutants display higher progeny numbers on days 4-6, but produced slightly fewer total progeny. *unc-25* encodes a neuronal-specific glutamic acid decarboxylase, which is required for GABA synthesis. These results indicate that GABergic signaling regulates egg-laying; this is in agreement with previous observations by Tanis et al. (2009) that muscimol, a selective agonist for GABA_*A*_ receptors, inhibits egg-laying similar to a mutation of the inhibitory *unc-49* GABA_*A*_ receptor. This is likely due to the fact that *C. elegans* vulval muscles receive synaptic input from a number of different neurons, including GABAergic VD neurons, and muscle contractions are required for egg-laying (Cermak et al., 2020).

## Environment: Temperature and Food Source

Reproduction and reproductive aging in *C. elegans* are influenced by the environment. Standard laboratory conditions for culturing *C. elegans* and measuring reproduction are nematode growth medium (NGM) agar dishes with a lawn of *E. coli* bacteria as a food source at 20°C. Reducing the temperature to 15°C delays reproductive aging (Figure 7B and Table 2), whereas increasing the temperature to 25°C accelerates reproductive aging (Hughes et al., 2007). There are two main theories for these temperature effects: (1) Both heat and cold are perceived as stresses, and the activation of stress response pathways modifies the reproductive schedule. (2) *C. elegans* are poikilotherm animals, and the rate of metabolic reactions responds to temperature, resulting in changes to the reproductive schedule. Both of these theories may be true, and it is difficult to distinguish these effects.

The most common *E. coli* strains used as a food source for *C. elegans* are OP50 or HT115 for classic developmental biology assays or RNAi experiments, respectively (Conte et al., 2015). Culturing *C. elegans* on different bacterial strains or species as a food source has been reported to cause a number of effects, which have been previously reviewed (Darby, 2005; Kim, 2013; Kim and Flavell, 2020; Poupet et al., 2020). The effect of different bacterial species on reproductive aging, however, is limited to a small number of recent studies (Table 2). Madhu et al. (2019) reported that wild-type *C. elegans* egg-laying curves changed when cultured on *S. marcescens* or *S. epidermidisi. S. marcescens* decreased the number of eggs laid at the peak and did not affect reproductive aging. By contrast, *S. epidermidis* extended the reproductive span significantly, with more eggs laid on days 5–11. The total brood size was slightly, but not significantly, decreased from ∼215 to ∼175 on average (Madhu et al., 2019).

In self-fertile hermaphrodites, changing the *E. coli* food source from OP50 to IAI1 or F11 increased the peak progeny number, while the total progeny number remained unchanged (Baeriswyl et al., 2010). Self-fertile hermaphrodites cultured on *Comamonas* sp., *Bacillus megaterium* or *E. coli* strains HB101, HT115, or MG1655 showed reduced reproductive lifespans compared to OP50 (Sowa et al., 2015). Furthermore, culturing hermaphrodites on HB101 reduces their reproductive success after exposure to males late in life (day 10/11) (Sowa et al., 2015). The authors suggest links between olfactory sensation, neuroendocrine signaling, and reproductive timing. Stuhr and Curran (2020) also reported that hermaphrodites cultured on *E. coli* OP50, HT115, and HB101 have the same number of total progeny. However, they found the shape of the progeny production curves is slightly altered. For example, HT115 and HB101 increase peak progeny numbers on day 2 and decrease progeny number on day 3 compared to OP50. By contrast, culture on *Methylobacterium braciatum* and *Sphingomonas aquatilis* significantly decreased total progeny number. While changing the bacterial diet can have striking effects, these experiments can be difficult to interpret because bacteria influence *C. elegans* in at least two ways. First, the bacteria are a complex set of chemical nutrients, and different bacterial strains and species change the amount and ratio of many nutrients at the same time. Second, bacteria have a spectrum of pathogenicity for *C. elegans*, and the standard *E. coli* OP50 strain is mildly pathogenic. Thus, changing bacteria can simultaneously affect pathogenesis, immune response, bacterial avoidance behavior, and nutrition.

## Ecological and Evolutionary Consequences of Reproductive Aging

A large amount of thought and theory have been expended to understand why animals age, and in particular why the reproductive system ages. Because reproductive aging limits individual progeny production, it is often considered a negative trait. Many have argued that evolution will select for animals that make more progeny for a longer period of time, and that animals that make less progeny for a shorter period of time will not compete successfully and will not pass on their genes. Self-fertile hermaphrodites such as *C. elegans* add additional complexity to these questions. Hodgkin and Barnes (1991) analyzed a mutant hermaphrodite that produces more male sperm before switching to oocyte production. These hermpahrodites can make a larger number of self-progeny than wild-type hermaphrodites, but they are delayed in generating the first egg compared to wild type. Thus, *C. elegans* hermaphrodites confront a tradeoff between time to first self-fertile egg laid and the total number of self-fertile eggs it can eventually lay. While observational and interventional studies of *C. elegans* cannot directly answer these teleological or “why” questions, the experimental power of the system makes it well suited to testing some of the predictions of these theories. Here we describe some prominent theories of why somatic and reproductive aging occurs, and consider how the results of studying *C. elegans* bear on these ideas.

To begin, it is worth considering a central conundrum of reproductive aging – if reproduction is the purpose of animal life, and evolution selects for greater levels of reproduction, then the intuitive prediction is that animals would reproduce up until the time they die – that reproduction would be the last system to fail, so that an individual could make as many progeny as possible while it still survived. *C. elegans* is ideal for testing this prediction, and the answer is clear. As a result of reproductive aging, germline function and reproductive capacity decline before somatic aging compromises life support systems (Figures 1B,C). Therefore, many hermaphrodites spend more than a third of the lifespan as post reproductive adults (Pickett et al., 2013). This is not simply a trait of worms – human females and many other animals display substantial post reproductive life spans. While it has been argued that humans may have a post reproductive lifespan to serve as grandparents, this does not apply to *C. elegans* or many other species that do not provide maternal care. Thus, to explain the pattern of reproductive aging observed in *C. elegans*, a theory should explain why reproduction fails before life support systems.

The theory of aging proposed by Medawar in a lecture delivered in 1952 has a prominent role in the field. Medawar proposed that the evolution of aging is driven by extrinsic death that gradually reduces the number of animals in a birth cohort. As the number of animals decreases, evolution by natural selection cannot effectively select for greater longevity. This is undoubtedly true in the limiting case – evolution by natural selection cannot promote additional longevity after all the animals in a cohort have died from extrinsic mortality. Because “the force of natural selection weakens with increasing age” (Medawar, 1952), deleterious traits or harmful alleles can accumulate, resulting in age-related pathologies or, simply, aging. According to this theory, reproductive aging developed in the “shadow of selection” of extrinsic mortality. In other words, reproductive aging is a deleterious trait, and high levels of progeny production with life-long reproductive capacity would be the favorable trait, but extrinsic death prevents natural selection from promoting further reproductive longevity.

A few years later, Williams extended this theory by proposing that selective forces favor early reproduction with the byproduct being antagonistically pleiotropic genes that result in the decline of reproduction later in life (Williams, 1957). Here Williams recognized the centrality of reproductive aging, which is why studies of reproductive aging are the key to evaluating these evolutionary theories. In addition, Williams postulated that “there should be little or no post reproductive period” (Gaillard and Lemaître, 2017). This prediction of the antagonistic pleiotropy theory is clearly not supported by studies of *C. elegans* or many other species. Both theories were founded on the assumption that age-related declines are a result of protected life in captivity, and that animals in the wild die only of extrinsic factors. Over the last decades, field studies reported many different species that experience age-related declines in the wild (Nussey et al., 2013). Thus, this assumption of these theories is not supported. The antagonistic pleiotropy theory is widely discussed because it predicts that decreasing early reproduction can cause an extension of the reproductive span. This pattern has frequently been observed in *C. elegans*. For example, mutations of *daf-2* and dietary restriction both reduce early progeny production and increase late progeny production. While these observations conform to a prediction of the antagonistic pleiotropy theory, they are also consistent with other theories. At this time, the most concerning aspect of these theories is that they do not appear to make predictions that enable the theories to be critically tested and falsified. Both Medawar and Williams were theorists, and they provided no data to evaluate these ideas. While experimental observations have been made that are consistent with these theories, there are also data that are not consistent, and these “flies in the ointment” are often addressed by theory defense. For example, if mutation #1 causes a decrease in early progeny production and an increase in late progeny production, then it supports the theory. If mutation #2 does not cause a decrease in early progeny production and yet still causes an increase in late progeny production, then it does not support the theory. However, theory defense could argue that mutation #2 may reduce early progeny production under some alternative environmental condition, and the fact that it was not observed in one or more conditions that were tested does not rule out the possibility that it would be observed in some condition. Thus, these data would not be accepted as falsifying the theory. It begins to appear that these theories have left the realm of hypothesis and entered the realm of belief.

Kirkwood’s disposable soma theory of aging, a modification of William’s antagonistic pleiotropy theory, builds on the assumption that “natural selection trades late survival for enhanced early fecundity” (Kirkwood and Rose, 1991) and explains aging by allocation of resources to reproduction earlier in life (Kirkwood et al., 2017). The occurrence of aging in the wild is in agreements with this theory- it would be the result of an optimal distribution key that only allocates a certain amount of energy to maintenance with the consequence of an optimal aging rate and lifespan (Kowald and Kirkwood, 2015). While this theory can explain somatic aging and lifespan, it is more complicated to apply to the data of reproductive aging in *C. elegans*. Is the early functional decline of the germ line followed by the reproductive capacity decline a result of misallocation of resources to maintenance of the reproductive system? While increased progeny production has clear trade-offs in many species due to intensive maternal care and limited resources, the trade-offs for reproductive longevity in *C. elegans* are less obvious. It also seems difficult to find the connection between resource allocation and the heterogeneous reproductive aging pattern that were reviewed here (Tables 2, 3 and Supplementary Table 1). Furthermore, Hsin and Kenyon (2009) showed that ablation of the entire gonad (germ line and somatic gonad) does not extend lifespan in *C. elegans*. Taken at face value, this result directly contradicts the disposable soma theory.

**FIGURE 9:**
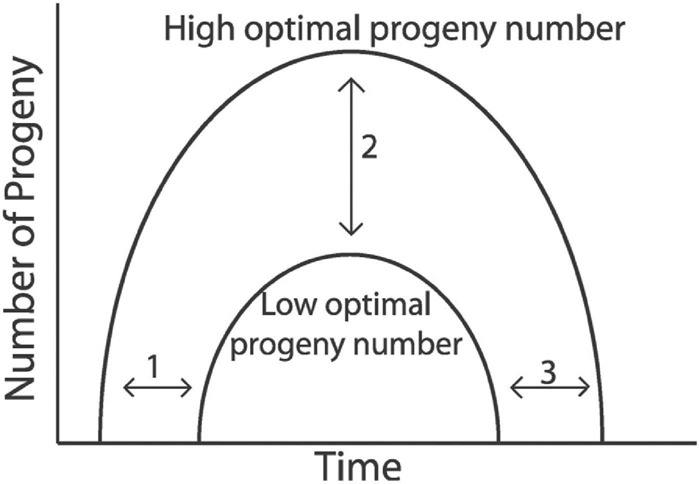
Optimal progeny number theory. The schematic of hypothetical progeny production curves display how progeny number can be controlled: (1) timing of onset of reproduction, (2) quantity of progeny, and (3) timing of cessation of reproduction or reproductive aging. Thus, reproductive aging is one of the critical inputs that controls progeny number. According to the theory, there is an optimal progeny number that stabilizes population dynamics and support long-term survival of populations. Therefore, reproductive aging can be an advantageous trait that might be sculpted by evolution. Adapted from Hughes et al. (2007).

All of these theories are based on the assumption that reproductive aging is a deleterious trait. By contrast, Hughes et al. (2007) proposed the optimal progeny number theory based on the assumption that reproductive aging is an advantageous trait (Figure 9; Hughes et al., 2007). According to this theory, reproductive aging is an adaptive trait that promotes reproductive success in the context of populations and their long-term survival in ecosystems with finite resources. An optimal progeny number can limit the population from growing too large in times of rich food availability and exceeding the carrying capacity of the environment. Populations that exceed carrying capacity enter a phase of starvation and instability characterized by boom and bust cycles and the threat of extinction. In contrast, reproductive restraint can stabilize population fluctuations and be advantageous for population survival in the long-term. The optimal progeny theory explains why reproductive aging declines before somatic aging – the central conundrum of this field. To achieve the optimal progeny production curve, the somatic tissues were selected for durability so they do not become the rate limiting step in reproduction. The optimal progeny number theory predicts the existence of mutations that decrease early reproduction and extend late reproduction. According to this theory, such genes promote early reproduction, which is adaptive, and they promote the age-related decline of reproduction, which is also adaptive. Thus, this theory explains the same observations that support the antagonistic pleiotropy theory without invoking genetic tradeoffs.

The key prediction of the optimal progeny number theory is that hermaphrodites that produce fewer progeny can be selected for by evolution because they stabilize population dynamics. Thus, to test this theory requires a new level of analysis -determining how the traits of individuals interact with population dynamics. In practice, this will require analyzing reproductive aging in the context of populations and ecosystems in the laboratory. Such an approach would make it possible to directly measure how different progeny production curves and progeny numbers affect population fluctuations over many generations in different environments. Most importantly, it would bridge the gap between the scarce data available from *C. elegans* in the wild with widespread data from individual animal measurements in the laboratory. This is an important future goal for the field, and advances in computational modeling make it seem achievable. Recently, Galimov and Gems (2020) reported a computational simulation that examined the role of aging during the population dynamics of a *C. elegans* colony. Future work in this area is likely to deliver important answers to fundamental questions in the field.

## Conclusion

(1) Detailed descriptions of age-related morphological changes in the *C. elegans* germ line are now available. They began with gross morphology determined by DIC optics, and have now been extended to measurements of the number, cell cycle duration, and rate of meiotic progression of the germ-line stem cells. These studies point to the distal germ line as the source of early degenerative change, which results in reduced egg-laying with a two-day delay (Figure 6E). The major challenge for future studies will be to elucidate the mechanism that controls this rapid and early decline. Because this decline occurs rapidly and in every animal, it seems unlikely to be caused by stochastic damage from the environment, which would be expected to be variable between animals. Rather, it appears to be caused by processes encoded in the genomes or environmental factors that affect every individual. Deciphering the mechanism of this population-wide decline would be a major achievement.
(2) Many genetic, pharmacological, and environmental factors that delay reproductive aging have now been identified. The challenge is to understand the chain of events that leads from the intervention to extended reproductive function. In most cases, little is currently known. One limitation of these studies is that the reported data are difficult to compare due to heterogeneity in methods and selective reporting of time points. Some studies only provided cross sectional data and reported progeny numbers for only a single day. To achieve a more standard and unified analysis, we suggest that future studies of reproductive aging interventions in *C. elegans* incorporate the following approaches: (1) hermaphrodites should be briefly mated with males (24–48 h) to avoid sperm depletion; (2) a complete progeny production curve should be presented, so that it is possible to determine total reproductive span, reproductive span after peak, peak progeny number, and total progeny number. An example is provided by Hughes et al. (2007). This unified protocol would help to compare data and to gain a deeper understanding of reproductive aging plasticity.
(3) It is still unclear why reproduction declines before the soma deteriorates in *C. elegans*. Future studies are needed to address this essential question and the related question of why *C. elegans* has a long post reproductive lifespan.
(4) Reproductive aging in *C. elegans* has only been studied in laboratory conditions where animals are relatively isolated and provided with an excess of bacterial food. While field studies in the wild are difficult to conduct due to the small size and subterranean lifestyle of these worms, experiments are needed to understand reproductive aging in the context of populations. A population-level approach can answer important questions such as (i) Is the post reproductive lifespan observed under optimal food conditions also present when worms are cultured in a population? (ii) How frequent is matricidal hatching in a population, and how does it affect population dynamics, (iii) Is there an optimal progeny number that can stabilize population dynamics, and how can reproductive restraint be maintained if mutants arise that make additional progeny? (iv) Can reproductive aging be adaptive, and if so in which conditions?
(5) Although *C. elegans* and humans have obvious differences, they also share striking similarities. In the case of reproductive aging, more information about the process in both worms and humans is necessary to fully evaluate the relevance of *C. elegans* as a model system. Several interesting parallels have emerged thus far: (1) *C. elegans* hermaphrodites and human females both display a substantial post-reproductive lifespan. (2) Oocyte quality displays age-related declines in both systems. (3) The same molecular pathways can control the rate of somatic aging in both systems, such as insulin/IGF-1 pathway and dietary restriction, and these similarities might extend to reproductive aging. If the systems share an underlying biology, then the experimental power of *C. elegans* is likely to accelerate the understanding of human reproductive aging.

## Author Contributions

AS, FP, BME, ZK, and KK provided scientific input and wrote the manuscript. All authors contributed to the article and approved the submitted version.

## Conflict of Interest

The authors declare that the research was conducted in the absence of any commercial or financial relationships that could be construed as a potential conflict of interest.

## Publisher’s Note

All claims expressed in this article are solely those of the authors and do not necessarily represent those of their affiliated organizations, or those of the publisher, the editors and the reviewers. Any product that may be evaluated in this article, or claim that may be made by its manufacturer, is not guaranteed or endorsed by the publisher.
