# Supplementary material for: Reproductive Aging in Caenorhabditis elegans: From Molecules to Ecology
Source: Front Cell Dev Biol. 2021 Sep 16;9:718522. doi: 10.3389/fcell.2021.718522 (PMC8481778; doi:10.3389/fcell.2021.718522)
Supplement: Supplementary file 1 [file Table_1.pdf]

Supplementary Table 1. Genetic interventions that modulate reproductive aging in *C. elegans*.

Intervention[1]		Longitudinal experiments[2]/ Male mated[3]	Reproductive span to peak (days)[4]	Reproductive span after peak (days)[5]	Total reproductive span (days)[6]	Peak progeny number[7]	Total progeny number[8]	Matricidal hatching[9]	Morphological phenotypes[10]	Reference
**TGF-β dauer and Sma/Mab pathway**	*daf-7 (e1372)*	yes/no	+/-	↑	↑	↓	ND	↑	ND	(Luo et al., 2009)
	daf-1 (m40)	yes/no	ND	ND	↑	ND	ND	ND	ND	(Luo et al., 2009)
	daf-14 (m77)	yes/no	+/-	↑	↑	↓	ND	ND	ND	(Luo et al., 2009)
	daf-4 (e1364)	yes/no	↑	↑	↑	↓	↓	↑	ND	(Luo et al., 2009)
	daf-3 (mgDf90)	yes/no	+/-	↑	↑	↓	ND	ND	ND	(Luo et al., 2009)
	sma-2 (e502)	yes/both	↑(only self-fertile)	↑	↑ (both mated and self-fertile)	↓	↓	↑	ND	(Luo et al., 2009)

	sma-2 RNAi	yes/both	ND	ND	↑(both mated and self-fertile)	ND	ND	ND	ND	(Luo et al., 2009)(Luo et al., 2010)
	sma-4 (e729)	yes/no	ND	↑ but difficult to measure due to matricidal hatching	not determined due to matricidal hatching	ND	ND	↑	ND	(Luo et al., 2009)
	sma-3 (wk20)	yes/both	ND	ND	↑ (both mated and self-fertile)	ND	ND	↑	ND	(Luo et al., 2009)
	sma-3 (wk28)	yes/no	ND	ND	↑	ND	ND	↑	ND	(Luo et al., 2009)(Luo et al., 2010)
	sma-3 (wk30)	yes/no	ND	ND	↑	ND	ND	ND	ND	(Templeman et al., 2020)
	dbl-1 (nk3)	yes/both*, no**,***	+/- **or ↑***	↑**,***	↑(both mated and self-fertile)	↓**	↓**	↑***	ND	(Luo et al., 2009*; Madhu et al., 2019**; de Lucas et al., 2021***)
	sma-9 (wk55)	yes/yes	+/-	↑	↑	↓	↓	↑	ND	(Luo et al., 2009)
	sma-9 (qc3)	yes/yes	↑	↑	↑	ND	↓	ND	ND	(Luo et al., 2009)
	crh-1 (n3450)	yes/yes	ND	ND	↑	ND	ND	ND	ND	(Templeman et al., 2020)

	crh-1 (n3315)	yes/yes	ND	↑	↑	ND	ND	ND	significant improvement in the morphology of aging oocytes	(Templeman et al., 2020)
	sma-10 (ok2224)	yes/no	+/-	↑	↑	↓	↓	↓	ND	(de Lucas et al., 2021)
**Hedgehog related**	*wrt-10* over expression (hypodermis)	yes/yes	ND	ND	↑	ND	ND	ND	delay of oocyte quality deterioration	(Templeman et al., 2020)
**Insulin/IGF-1 signaling pathway**	*age-1(hx546)*	yes/yes day1	+/-	ND	+/-	↓	↓	ND	ND	(Hughes et al., 2007)
	daf-2 (e1370)	yes/yes day1	+/-	ND	+/-	↓	↓	ND	ND	(Hughes et al., 2007)
	daf-2 (m41)	yes/yes day1	+/-	ND	↓	↓	↓	ND	ND	(Hughes et al., 2007)
	daf-2 (RNAi)	yes/no	+/-	↑	↑	+/-	ND	ND	ND	(Wang et al., 2014)
	daf-16(mu86):daf-2(e1370)	yes/yes day1	+/-	ND	↑	+/-	+/-	ND	ND	(Hughes et al., 2007)

	daf-16(mu86)	yes/yes day1	+/-	ND	+/-	+/-	↓	ND	ND	(Hughes et al., 2007)
	daf-16(m26)	yes/yes day1	+/-	ND	+/-	+/-	↓	ND	ND	(Hughes et al., 2007)
	sucg-1	yes/no	ND	ND	↑	ND	ND	ND	ND	(Wang et al., 2014)
	F36F2.2	yes/no	ND	ND	↑	ND	+/-	ND	ND	(Wang et al., 2014)
	nhr-85	yes/no	ND	ND	↑	ND	ND	ND	ND	(Wang et al., 2014)
**Sodium homeostasis**	*nhx-2* (RNAi)	yes/no	+/-	↑	↑	↓	ND	ND	ND	(Wang et al., 2014)
	sgk-1 (RNAi)	yes/no	↑	↑	↑	↓	ND	ND	ND	(Wang et al., 2014)
**Dietary restriction**	*eat-2(ad465)*	yes/yes day1	+/-	↑	↑	↓	↓	↓	ND	(Hughes et al., 2007)
	eat-2 (ad465)	yes/both	↑	↑	↑(both mated and self-fertile)	↓	↓	↑	ND	(Luo et al., 2009)
	eat-2 (RNAi)	yes/both	↑	↑	↑(both mated and self-fertile)	↓	↓	↑	ND	(Luo et al., 2009)

	eat-2(ad1116)	yes/no	↑	↑	↑	↓	↓	ND	ND	(Crawford et al., 2007)
	phm-2(am117)	yes/yes	↑	↑	↑	↓	↓	↑	ND	(Hughes et al., 2011; Kumar et al., 2019)
**unc-25 (GABA synthesis)**	*unc-25 (e156)*	yes/no	↑	↑	↑	↓	↓	ND	ND	(Cermak et al., 2020)
	unc-25 (n2324)	yes/no	↑	↑	↑	↓	↓	ND	ND	(Cermak et al., 2020)
**Mitochondrial genes**	*clk-1(qm30)*	yes/yes day1	↑	ND	+/-	↓	↓	ND	ND	(Hughes et al., 2007)
	isp-1(qm150)	yes/yes day1	↑	ND	+/-	↓	↓	ND	ND	(Hughes et al., 2007)
dhcr-24 (F52H2.6 RNAi)		yes/no	+/-	+/-	+/-	↑	↑	ND	ND	(Kim et al., 2018)
moma-1 (RNAi)		yes/no	ND	ND	↑	ND	ND	ND	ND	(Wang et al., 2014)
F37C4.8 (RNAi)		yes/no	ND	ND	↑	ND	ND	ND	ND	(Wang et al., 2014)
Y38H6C.21 (RNAi)		yes/no	ND	ND	↑	ND	ND	ND	ND	(Wang et al., 2014)

R07H5.9 (RNAi)	yes/no	ND	ND	↑	ND	ND	ND	ND	(Wang et al., 2014)
VC27A7L.1 (RNAi)	yes/no	ND	ND	↑	ND	ND	ND	ND	(Wang et al., 2014)
ilys-3 (RNAi)	yes/no	ND	ND	↑	ND	↓	ND	ND	(Wang et al., 2014)
sucl-2 (RNAi)	yes/no	↑	↑	↑	↓	ND	ND	ND	(Wang et al., 2014)
oac-16 (RNAi)	yes/no	ND	ND	↑	ND	ND	ND	ND	(Wang et al., 2014)
adal-1 (RNAi)	yes/no	ND	ND	↑	ND	+/-	ND	ND	(Wang et al., 2014)
F25H8.1 (RNAi)	yes/no	ND	ND	↑	ND	+/-	ND	ND	(Wang et al., 2014)
daf-3 (RNAi)	yes/no	ND	ND	↑	ND	+/-	ND	ND	(Wang et al., 2014)
rskn-1 (RNAi)	yes/no	ND	ND	↑	ND	ND	ND	ND	(Wang et al., 2014)

igdb-2 (RNAi)	yes/no	ND	ND	↑	ND	ND	ND	ND	(Wang et al., 2014)
F20B10.3 (RNAi)	yes/no	ND	ND	↑	ND	+/-	ND	ND	(Wang et al., 2014)
T04B2.1 (RNAi)	yes/no	ND	ND	↑	ND	+/-	+/-	ND	(Wang et al., 2014)
Y58A7A.1 *(RNAi)*	yes/no	ND	ND	↑	ND	ND	ND	ND	(Wang et al., 2014)
cox-18 (RNAi)	yes/no	ND	ND	↑	ND	ND	ND	ND	(Wang et al., 2014)
F54E2.1 (RNAi)	yes/no	ND	ND	↑	ND	ND	ND	ND	(Wang et al., 2014)
hmr-1 (RNAi)	yes/no	ND	ND	↑	ND	ND	ND	ND	(Wang et al., 2014)
unk-1 (RNAi)	yes/no	ND	ND	↑	ND	ND	ND	ND	(Wang et al., 2014)

[1]Intervention: gene with mutant allele or RNAi.
[2]Longitudinal experiment: the same individual animal is observed at sequential time points. yes: measured in a longitudinal experiment, no: measured in a cross-sectional experiment.
[3]Male mated: yes- hermaphrodites were exposed to males, with adult day of exposure specified, no: self-fertile hermaphrodites.

[4]Reproductive span to peak: time from adult day 0 until day of peak egg-laying (Figure 1).
[5]Reproductive span after peak: time from day of peak egg-laying until last day of egg-laying (Figure 1).
[6]Total reproductive span: time from adult day 0 until last day of egg-laying (Figure 1).
[7]Peak progeny number: Number of eggs laid on day of peak progeny production (Figure 1).
[8]Total progeny number: Total number of eggs laid (Figure 1).
[9]Matricidal hatching: If mature hermaphrodites cannot deposit eggs into the environment, then eggs hatch inside the hermaphrodite, and larvae feed on the parent resulting in death.
[10]Morphological phenotypes: interventions may affect age-related changes in gonad morphology.

[4-9] ↑: increase; ↓: decrease; +/-: no change; ND: not determined.

Literature

Cermak, N., Yu, S. K., Clark, R., Huang, Y. C., Baskoylu, S. N., and Flavell, S. W. (2020). Whole-organism behavioral profiling reveals a role for dopamine in statedependent motor program coupling in C. Elegans. *Elife* 9, 1–34. doi:10.7554/eLife.57093.

Crawford, D., Libina, N., and Kenyon, C. (2007). Caenorhabditis elegans integrates food and reproductive signals in lifespan determination. *Aging Cell* 6, 715–721. doi:10.1111/j.1474-9726.2007.00327.x.

de Lucas, M. P., Jiménez, M., Sánchez-Pavón, P., Sáez, A. G., and Lozano, E. (2021). SMA-10 is a non-canonical member of the TGF-β sma/mab pathway and immunity regulator via the DAF-2 insulin receptor in caenorhabditis elegans. *Int. J. Mol. Sci.* 22, 1–13. doi:10.3390/ijms22020638.

Hughes, S. E., Evason, K., Xiong, C., and Kornfeld, K. (2007). Genetic and pharmacological factors that influence reproductive aging in nematodes. *PLoS Genet.* 3, 0254–0265. doi:10.1371/journal.pgen.0030025.

Hughes, S. E., Huang, C., and Kornfeld, K. (2011). Identification of mutations that delay somatic or reproductive aging of Caenorhabditis elegans. *Genetics* 189, 341–356. doi:10.1534/genetics.111.130450.

Kim, Y., Park, Y., Hwang, J., and Kwack, K. (2018). Comparative genomic analysis of the human and nematode caenorhabditis elegans uncovers potential reproductive genes and disease associations in humans. *Physiol. Genomics* 50, 1002–1014. doi:10.1152/physiolgenomics.00063.2018.

Kumar, S., Egan, B. M., Kocsisova, Z., Schneider, D. L., Murphy, J. T., Diwan, A., et al. (2019). Lifespan Extension in C. elegans

Caused by Bacterial Colonization of the Intestine and Subsequent Activation of an Innate Immune Response. *Dev. Cell* 49, 100-117.e6. doi:10.1016/j.devcel.2019.03.010.

Luo, S., Kleemann, G. A., Ashraf, J. M., Shaw, W. M., and Murphy, C. T. (2010). TGF-β and Insulin Signaling Regulate Reproductive Aging via Oocyte and Germline Quality Maintenance. *Cell* 143, 299–312. doi:10.1016/j.cell.2010.09.013.

Luo, S., Shaw, W. M., Ashraf, J., and Murphy, C. T. (2009). TGF-ß Sma/Mab Signaling Mutations Uncouple Reproductive Aging from Somatic Aging. *PLoS Genet.* 5, e1000789. doi:10.1371/journal.pgen.1000789.

Madhu, B., Salazar, A., and Gumienny, T. (2019). Caenorhabditis elegans egg-laying and brood-size changes upon exposure to Serratia marcescens and Staphylococcus epidermidis are independent of DBL-1 signaling. *microPublication Biol.* 2019. doi:10.17912/2r51-b476.

Templeman, N. M., Cota, V., Keyes, W., Kaletsky, R., and Murphy, C. T. (2020). CREB Non-autonomously Controls Reproductive Aging through Hedgehog/Patched Signaling. *Dev. Cell* 54, 92-105.e5. doi:10.1016/j.devcel.2020.05.023.

Wang, M. C., Oakley, H. D., Carr, C. E., Sowa, J. N., and Ruvkun, G. (2014). Gene Pathways That Delay Caenorhabditis elegans Reproductive Senescence. *PLoS Genet.* 10, e1004752. doi:10.1371/journal.pgen.1004752.
